# Predicting ultrahigh risk multiple myeloma by molecular profiling: an analysis of newly diagnosed transplant eligible myeloma XI trial patients

**DOI:** 10.1038/s41375-020-0750-z

**Published:** 2020-03-11

**Authors:** Vallari Shah, Amy L. Sherborne, David C. Johnson, Sidra Ellis, Amy Price, Farzana Chowdhury, Jack Kendall, Matthew W. Jenner, Mark T. Drayson, Roger G. Owen, Walter M. Gregory, Gareth J. Morgan, Faith E. Davies, Gordon Cook, David A. Cairns, Richard S. Houlston, Graham Jackson, Martin F. Kaiser, on behalf of NCRI Haematology Clinical Studies Group, Peter Hillman, Lesley Anderson, Stephen O’Brien, Jim Cavet, Oliver Ottman, Alan Chant, Alasdair Rankin, Gordon Cook, Clare Rowntree, Charles Craddock, Anna Schuh, Lavinia Davey, Shamyla Siddique, Walter Gregory, Simon Stanworth, Sally Killick, Simon Watt, Amy Kirkwood, Kwee Yong, Dragana Milojkovic, Thomas Fox, Adam Mead, Gillian Horne, Gillian Murphy, Kikkeri Naresh

**Affiliations:** 1 Division of Molecular Pathology, The Institute of Cancer Research, London, UK; 2 Department of Haematology, University Hospital Southampton NHS Foundation Trust, Southampton, UK; 3 Institute of Immunology and Immunotherapy, University of Birmingham, Birmingham, UK; 4 Haematological Malignancy Diagnostic Service, St. James’s University Hospital, Leeds, UK; 5 Clinical Trials Research Unit, Leeds Institute of Clinical Trials Research, Leeds, UK; 6 Perlmutter Cancer Center, NYU Langone Health, New York, NY USA; 7 Leeds Institute of Cancer and Pathology, University of Leeds, Leeds, UK; 8 Molecular and Population Genetics, The Institute of Cancer Research, London, UK; 9 Department of Haematology, University of Newcastle, Newcastle Upon Tyne, UK

**Keywords:** Myeloma, Cancer genomics, Cancer genetics

The prognosis for newly diagnosed multiple myeloma (NDMM) has improved with the advent of new agents, but outcome in some patients remains very poor. Identifying patients with high-risk disease early opens up the prospect of stratified treatment [1–3].

Biomarkers including chromosomal aberrations t(4;14), t(14;16), and t(14;20) translocations, gain of 1q and deletion of 17p, detected by fluorescence in situ hybridization (FISH) or multiplex ligation-dependent probe amplification (MLPA) and qRT-PCR-based translocation detection, have been associated with adverse outcome and co-occurrence of ≥2 such aberrations (a double-hit) is predictive of especially aggressive MM [4, 5]. Multiple gene expression profiles (GEP) have been reported to be associated with outcome, but so far only EMC92 and UAMS GEP70 have been developed into validated clinical tests, marketed as SKY92 MMprofiler and MyPRS, respectively [6–9].

To examine the combined predictive value of high-risk chromosomal abnormalities and SKY92 risk GEP we studied 329 NDMM patients from the NCRI Myeloma XI trial (ISRCTN49407852) who received intensive therapy (Supplementary Table 1) and validated findings in Medical Research Council (MRC) Myeloma IX trial patients (Supplementary Methods) [10, 11]. In both cohorts of patients purified (>95%) CD138-positive tumor cells were immunomagnetically selected and DNA and RNA were extracted using QIAGEN (Hilden, Germany) Allprep kits. Chromosomal aberrations, including high-risk lesions t(4;14), t(14;16), t(14;20), gain(1q), and del(17p), were assessed using qRT-PCR (Thermo Fisher, Darford, UK) and MLPA (MRC Holland, Amsterdam, The Netherlands), as previously reported (Supplementary Methods) [4]. GEP risk status was determined on a diagnostic Affymetrix GeneChip 3000 Dx v2.0 system (Thermo Fisher) using the SKY92 MMProfiler (SkylineDx, Rotterdam, The Netherlands) (Supplementary Methods). Statistical analyses were performed in R (version 3.5.1) as detailed in Supplementary Methods.

MMprofiler assay results by SkylineDx identified 81 of the 329 Myeloma XI trial patients (24.6%) to have a SKY92 high-risk tumor signature (Supplementary Table 2). SKY92 high-risk patients had significantly shorter PFS (median 16.0 vs. 33.8 months; HR 2.6, 95% CI: 2.0–3.5; *P* = 4.1 × 10^−11^) and OS (median 36.7 months vs. not reached; HR 3.9, 95% CI: 2.7–5.7; *P* = 2.5 × 10^−13^) (Supplementary Fig. 1; Table 1), regardless of induction regimen and posttransplant randomization (Supplementary Figs. 2, 3; Supplementary Tables 3–5). Specifically, patients with SKY92 high-risk disease did not derive statistically significant benefit from lenalidomide single agent maintenance therapy (Fig. 1; Supplementary Fig. 3).

**Table 1:** Univariate and multivariate Cox proportional hazard survival analyses of genetic, gene expression, and clinical risk markers for PFS and OS for 329 representative Myeloma XI NDMM patients from induction randomization.

	Univariate analysis			Multivariate analysis	
*Progression free survival*			*Progression free survival*		
	HR (95% CI)	Wald *P*		HR (95% CI)	Wald *P*
SKY92 high-risk	2.6 (1.96–3.45)	4.08 × 10^−11^	SKY92 high-risk	2.14 (1.54–2.96)	**0.00000475**
Hyperdiploid	0.74 (0.57–0.95)	0.0198	Hyperdiploid	0.93 (0.7–1.24)	0.634
Adverse translocation	2.04 (1.53–2.72)	1.12 × 10^−06^	Adverse translocation	1.89 (1.36–2.62)	**0.00015**
Del(1p) [*CDKN2C*]	1.47 (1–2.18)	0.0514	Del(1p) [*CDKN2C*]	1.01 (0.65–1.56)	0.979
Del(17p) [*TP53*]	1.63 (1.09–2.42)	0.016	Del(17p) [*TP53*]	1.32 (0.87–2.0)	0.198
Gain(1q)	1.44 (1.11–1.88)	0.00634	Gain(1q)	0.88 (0.65–1.2)	0.425
Age	1.04 (1.02–1.06)	0.00012	Age	1.04 (1.02–1.06)	**0.000144**
Induction randomization	0.77 (0.59–0.99)	0.0417	Induction randomization	1.2 (0.92–1.55)	0.176
ISS	1.33 (1.12–1.58)	0.0012	ISS	1.13 (0.95–1.36)	0.176
			*n* = 328, events = 232		
*Overall survival*			*Overall survival*		
	HR (95% CI)	Wald *P*		HR (95% CI)	Wald *P*
SKY92 high-risk	3.94 (2.73–5.69)	2.54 × 10^−13^	SKY92 high-risk	2.72 (1.78–4.16)	**0.00000396**
Hyperdiploid	0.6 (0.42–0.87)	0.00717	Hyperdiploid	0.91 (0.6–1.37)	0.647
Adverse translocation	2.5 (1.72–3.64)	1.67 × 10^−06^	Adverse translocation	1.85 (1.19–2.88)	**0.0061**
Del(1p) [*CDKN2C*]	2.38 (1.49–3.79)	0.000271	Del(1p) [*CDKN2C*]	1.29 (0.76–2.2)	0.343
Del(17p) [*TP53*]	3.02 (1.87–4.87)	5.76 × 10^−06^	Del(17p) [*TP53*]	2.48 (1.48–4.17)	**0.000602**
Gain(1q)	2.39 (1.66–3.44)	2.98 × 10^−06^	Gain(1q)	1.3 (0.85–1.97)	0.222
Age	0.62 (0.43–0.9)	0.0113	Age	1.02 (0.99–1.05)	0.2
Induction randomization	0.62 (0.43–0.9)	0.0113	Induction randomization	1.31 (0.9–1.91)	0.153
ISS	1.38 (1.08–1.76)	0.0101	ISS	1.09 (0.84–1.43)	0.512
			*N* = 328, events = 117		

Statistically significant *P* < 0.05 values are in bold.

**Fig. 1:**
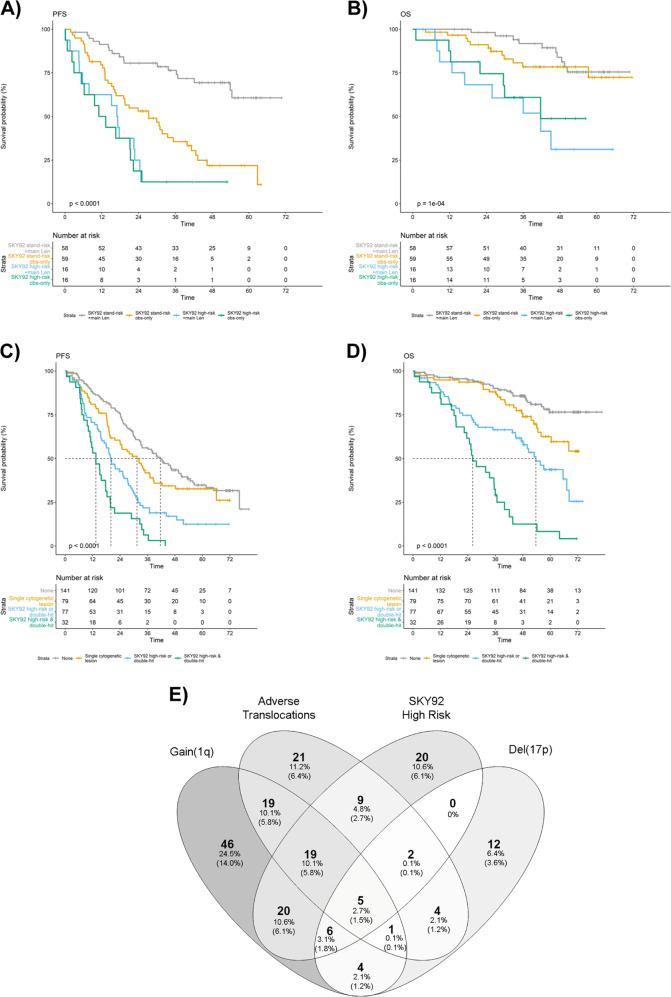
Patient outcome in context of GEP and chromosomal high-risk markers and their respective frequencies and distribution in Myeloma XI. Kaplan–Meier plot of Myeloma XI trial patients (*n* = 329) in context of SKY92 risk profiling for (**a**) PFS, (**b**) OS from maintenance randomization, with survival curves for patients randomized to lenalidomide or observation plotted separately. Log-rank *P* values displayed. **c**, **d** Kaplan–Meier plots of molecular risk groups defined by absence of any high-risk marker, presence of a single genetic risk marker, presence of either double-hit or SKY92 high-risk or combined double-hit and SKY92 high-risk for **c** PFS, **d** OS from induction randomization. **e** Venn diagram of patients with tumors positive for validated genetic risk markers adverse translocations, gain(1q), del(17p), SKY92 GEP high-risk. % is relative to 188 patients with high-risk lesions, (%) relative to all patients (*n* = 329) in the study. Frequency represented by gray color coding, with darker gray indicating higher frequency.

There was partial overlap between patients with GEP or chromosomal high-risk markers (Fig. 1, Supplementary Table 2), with 6.1% (20/329) of patients showing SKY92 positivity but absence of chromosomal high-risk markers. We analyzed prognostic association of molecular and clinical risk markers in a multivariable Cox proportional hazard model and found presence of SKY92 high-risk (HR 2.7, 95% CI: 1.8–4.2; *P* = 4.4 × 10^−6^), adverse translocations (HR 1.8, 95% CI: 1.2–2.9; *P* = 0.007), and del(17p) (HR 2.5, 95% CI: 1.5–4.1; *P* = 0.0007) to be independently associated with shorter OS and SKY92 high-risk (HR 2.1, 95% CI: 1.5–3.0; *P* = 4.8 × 10^−6^) and adverse translocations (HR 1.9, 95% CI: 1.4–2.6; *P* = 0.0002) with shorter PFS (Table 1). Results were similar when analyzing GEP risk status with the UAMS GEP70 signature: by multivariable analysis UAMS GEP70 high-risk (HR = 2.54; 95% CI: 1.56–4.13; *P* = 1.8 × 10^−4^), presence of del(17p) (HR = 2.22; 95% CI: 1.32–3.72; *P* = 0.0025), and adverse translocation (HR = 2.11; 95% CI: 1.35–3.28; *P* = 9.5 × 10^−4^) were independently associated with shorter OS. However, GEP70 was not independently associated with shorter PFS (Supplementary Table 6).

One hundred and sixty-one patient tumors carried no chromosomal high-risk marker, of which 20 (12%) were SKY92 high-risk. The presence SKY92 GEP high-risk in isolation was significantly associated with shorter PFS (HR = 3.18; 95% CI: 1.86–5.46; *P* = 2.6 × 10^−5^; median 15.8 vs. 41.7 months) and OS (HR = 2.42; 95% CI: 1.04–5.67; *P* = 0.04; estimated 4 year OS 55% vs. 86%; Supplementary Fig. 4; Supplementary Table 7).

We have previously demonstrated the adverse prognosis of double-hit tumors, defined by co-occurrence of ≥2 chromosomal high-risk markers [4]. SKY92 and double-hit were independently prognostic by multivariable analysis, with HRs 2.9 (95% CI: 1.9–4.2; *P* = 2.6 × 10^−7^) and 2.3 (95% CI: 1.5–3.6; *P* = 0.0002) for OS and HRs 2.0 (95% CI: 1.5–2.8; *P* = 6.8 × 10^−6^) and 1.6 (95% CI: 1.2–2.3; *P* = 0.005) for PFS, respectively (Supplementary Table 8). Results were consistent when PFS and OS were measured from maintenance randomization (Supplementary Table 8). We defined four risk groups combining predictive SKY92 and chromosomal high-risk markers: double-hit AND SKY92 (9.7% of pts), double-hit OR SKY92 (23.4% of pts), a single chromosomal high-risk marker (24.0% of pts), and no risk marker (42.9% of pts). Hazard ratios for OS were 11.0 (95% CI: 6.3–19.1; *P* < 2.2 × 10^−16^), 3.8 (95% CI: 2.3–6.3; *P* = 2 × 10^−7^), and 1.9 (95% CI: 1.1–3.3; *P* = 0.03) compared with those without risk markers, and HRs for PFS were 4.5 (95% CI: 3.0–6.9; *P* = 2.3 × 10^−12^), 2.3 (95% CI: 1.7–3.3; *P* = 4.4 × 10^−7^), and 1.3 (95% CI: 0.9–1.9; *P* = 0.118), respectively (Fig. 1; Supplementary Table 9). Results were consistent when PFS and OS were measured from time point of ASCT (Supplementary Fig. 5; Supplementary Table 9).

Of note, lenalidomide single agent maintenance markedly extended PFS in patients with a single chromosomal high-risk marker (HR 0.11; 95% CI: 0.03–0.41; *P* = 0.0001) or no risk marker (HR 0.26; 95% CI: 0.12–0.58; *P* = 0.001) when compared with observation. In contrast, those with SKY92 and/or double-hit (HR 0.67; 95% CI: 0.12–1.72, *P* = 0.24; HR 0.67; 95% CI: 0.32–1.37; *P* = 0.27, respectively) did not derive consistent benefit from lenalidomide maintenance (Supplementary Fig. 6).

Patients with combined double-hit and SKY92 high-risk status (9.7%) had poor survival outcomes: all patients (100%) progressed within 48 months from initial randomization and predicted OS at 48 months was 12.5% (Fig. 1). To confirm ultra-high-risk behavior of combined double-hit and SKY92 tumors in an independent trial, we analyzed 116 patients from the intensive, transplant treatment arm of MRC Myeloma IX. Eight (6.9%) patients showed double-hit and EMC92 ultra-high-risk; all patients progressed within 36 months and died within 48 months. Meta-analysis using a random-effect model showed a HR for OS of 6.0 (95% CI: 4.1–8.9; *P* = 4.8 × 10^−20^) and HR of 3.5 (95% CI: 2.5–4.9; *P* = 6.9 × 10^−13^) for PFS for patients with combined GEP and double-hit tumors (Supplementary Fig. 7).

After accounting for GEP and chromosomal high-risk status, ISS and serum LDH were not predictive of outcome (Supplementary Fig. 8; Supplementary Table 10). We found significant overlap of these clinical and molecular risk markers: frequency of ISS 3 was higher in SKY92 high-risk vs. non-high-risk (38% vs. 21%; *P* = 0.003; Supplementary Fig. 9) and in those with multiple chromosomal high-risk risk lesions (21.5% without vs. 28.6% with single hit vs. 43.8% with double-hit). Only 15.6% of double-hit tumor patients were ISS 1 at diagnosis (Supplementary Fig. 9), whereas 71% of patients with ISS 3 carried one or more chromosomal or SKY92 high-risk marker. Similarly, baseline LDH was higher in patients with SKY92 or double-hit tumors vs. those without (Supplementary Figs. 9, 10).

We furthermore interrogated a range of risk signatures beyond binary (high-risk/non-high-risk) clinical read-out. Quantitative risk scores were correlated for most clinical signatures, most markedly EMC92 and GEP70 (*r* = 0.79; *P* < 0.001) (Supplementary Fig. 11). The EMC92 score (*r* = 0.64, *P* < 0.001) as well as most others also correlated with the in vitro model derived Proliferation Index (Supplementary Fig. 11).

Extreme copy number abnormalities (CNAs; amplification (≥4 copies) or homozygous deletion) have recently been proposed as exclusive drivers of high-risk MM [12], prompting us to investigate the correlation of quantitative CNAs with GEP risk scores. Median EMC92 scores were higher in tumors with gain(1q) vs. those without (*P* = 2.1 × 10^−8^) but there was no difference between gain (three copies) and amplification of 1q (≥4 copies) (*P* = 0.56; Supplementary Fig. 12), with a wide range of GEP scores in the latter. Tumors with deletion 17p had significantly higher median EMC92 GEP scores than those without deletion. Homozygous del(17p) was rare (*n* = 2), as expected, not allowing for formal comparison (Supplementary Fig. 12). Tumors with high-risk translocations had on average higher EMC92 scores than those without (Supplementary Fig. 13).

Our results demonstrate the prospective prognostic validity of SKY92 profiling in the wider context as a means of identifying patients at diagnosis who have high-risk MM, and show the independent association of SKY92 and high-risk chromosomal aberrations with outcome [9]. Our results highlight the molecular diversity of MM and demonstrate that single time point combined GEP and chromosomal profiling at diagnosis can predict clinical outcome with significant precision, in line with recent findings across multiple solid cancers [13, 14].

We furthermore demonstrate that in context of combined SKY92 and chromosomal profiling, ISS and LDH are not independently predictive. This is perhaps not unexpected, since ISS and LDH are clinical surrogate markers for tumor proliferation, which is assessed by combined GEP and double-hit profiling. Our analysis was, however, limited to younger and fitter, transplant-eligible patients and clinical risk markers such as ISS may have greater and independent relevance in older or frailer patients [15].

Our results demonstrate that patients with double-hit or GEP high-risk status are unlikely to benefit from current treatment approaches, including single agent lenalidomide maintenance therapy. In such patients intensified ongoing therapy with combination agents may be beneficial [3]. Such an assertion will be prospectively assessed in clinical studies such as the risk stratified UK OPTIMUM (MUKnine) trial (NCT03188172).

In conclusion, our findings support the further adoption of molecular biomarkers to stratify NDMM patient therapy.

## Supplementary information

Supplementary Information
