# Supplementary material for: Predicting ultrahigh risk multiple myeloma by molecular profiling: an analysis of newly diagnosed transplant eligible myeloma XI trial patients
Source: Leukemia. 2020 Mar 11;34(11):3091–6. doi: 10.1038/s41375-020-0750-z (PMC7584474; doi:10.1038/s41375-020-0750-z)

SUPPLEMENTARY METHODS

Patients

We studied 329 NDMM patients enrolled in the intensive (transplant-eligible) treatment pathway of UK NCRI Myeloma XI (ISRCTN49407852), the first outcomes of which have been published recently.[1] Briefly, these patients were initially randomized to triplet induction based on thalidomide (CTD) or lenalidomide (CRD) in combination with cyclophosphamide and dexamethasone with treatment intensification with cyclophosphamide, bortezomib and dexamethasone (CVD) vs no intensification for insufficient responders (partial or minimal response) and intensification (CVD) for non-responders (stable or progressive disease). After high-dose melphalan (HDMEL) and autologous stem-cell transplantation (ASCT), patients were randomized to receive lenalidomide, lenalidomide plus vorinostat or observation. Maintenance treatment continued until progressive disease in the absence of toxicity (see Trial Flowcharts, below). The 329 patients were selected by the Clinical Trials Research Unit (CTRU) at the University of Leeds based on age, sex, induction treatment and maintenance treatment to reflect the overall trial population (Supplementary Table 1).

To validate study findings, we analyzed 116 transplant-eligible pathway patients from the MRC Myeloma IX trial (ISRCTN68454111) with chromosomal aberration and gene expression data (GSE15695). Myeloma IX trial outcomes have been previously reported.[2] Briefly, patients in this trial were randomized to receive induction with cyclophosphamide, vincristine, doxorubicin and dexamethasone (C-VAD) or CTD, followed by HDMEL and ASCT and a second randomization to thalidomide or observation (see Trial Flowcharts, below).

All patients provided written informed consent. The studies were approved by the national ethics review board (National Research Ethics Service, UK), institutional review boards of the participating centres, and the competent regulatory authority (Medicines and Healthcare Products Regulatory Agency, UK), and done according to the Declaration of Helsinki and the principles of Good Clinical Practice as espoused in the Medicines for Human Use (Clinical Trials) Regulations.

Translocation and copy number profiling

Recurrent immunoglobulin locus translocations including t(4;14), t(14;16), t(14;20), t(11;14) and t(6;14) were assessed by qRT-PCR as previously described.[3] Copy number aberrations were profiled using MLPA Salsa-P425 mix (MRC Holland, The Netherlands) and analyzed using Coffalyser software (MRC Holland) following manufacturer's instructions. Deletions and gain/amplification was called as previously described, hyperdiploidy was defined by gain of at least 2 of consensus chromosomes 5, 9 or 15.[3]

Gene expression profiling

GEP was performed on a diagnostic Affymetrix GeneChip 3000 Dx v2.0 system following the SKY92 MMProfiler (SkylineDx, The Netherlands) protocol. Anonymized GEP data was transferred, processed centrally by SkylineDx as per standard protocol and binary SKY92 risk status (High risk/Standard risk) reported back as part of a standard diagnostic report. For exploratory analyzes, data was normalized as described previously for the EMC92 score and batch corrected to HOVON gene expression arrays deposited at the NCBI-GEO repository (GSE19784).[4] Gene expression continuous scores for other risk signatures were calculated as previously described.[4-9]

Statistical analyses

Statistical analyses were performed in R (version 3.5.1) using sub-routines survival, metaphor and base R functions. Progression-free survival (PFS) was defined as time from induction or maintenance randomization or time of transplant to progression or death and overall survival (OS) as time from induction or maintenance randomization or time of transplant to death. Cox proportional hazards regression was used to estimate univariate and multivariable hazard ratios (HRs) and 95% confidence intervals (CI). Kaplan–Meier survival curves were generated and homogeneity between groups was assessed using the log-rank test. Association between categorical variables was examined using Fishers exact test and between continuous variables using the Wilcoxon signed-rank test or t-test for normally distributed variables. Correlation between continuous variables was calculated using Pearson product-moment correlation. A two-sided P-value of ≤ 0.05 was considered statistically significant.

TRIAL FLOWCHARTS

Trial outline NCRI Myeloma XI, intensive treatment pathway

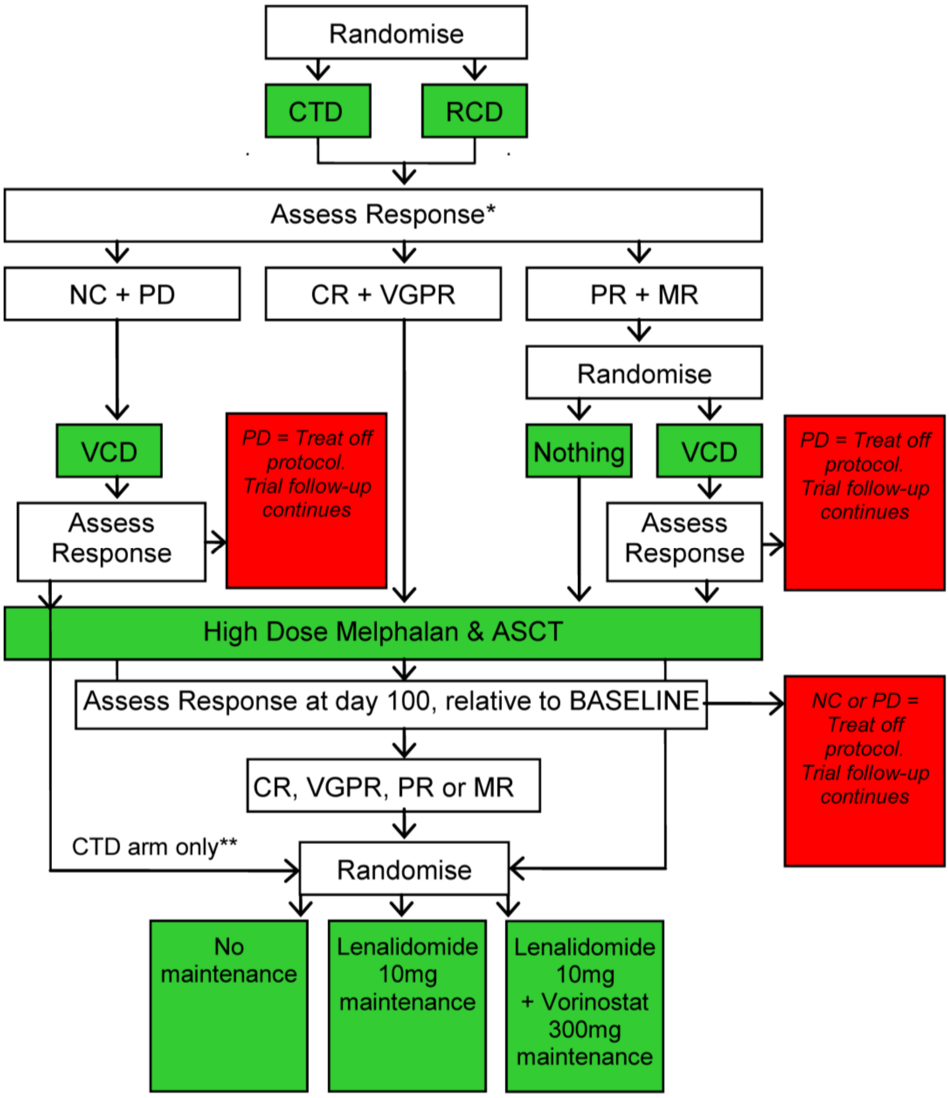

CONSORT diagram MRC Myeloma IX. Only patients from the intensive treatment pathway were included in the current analysis

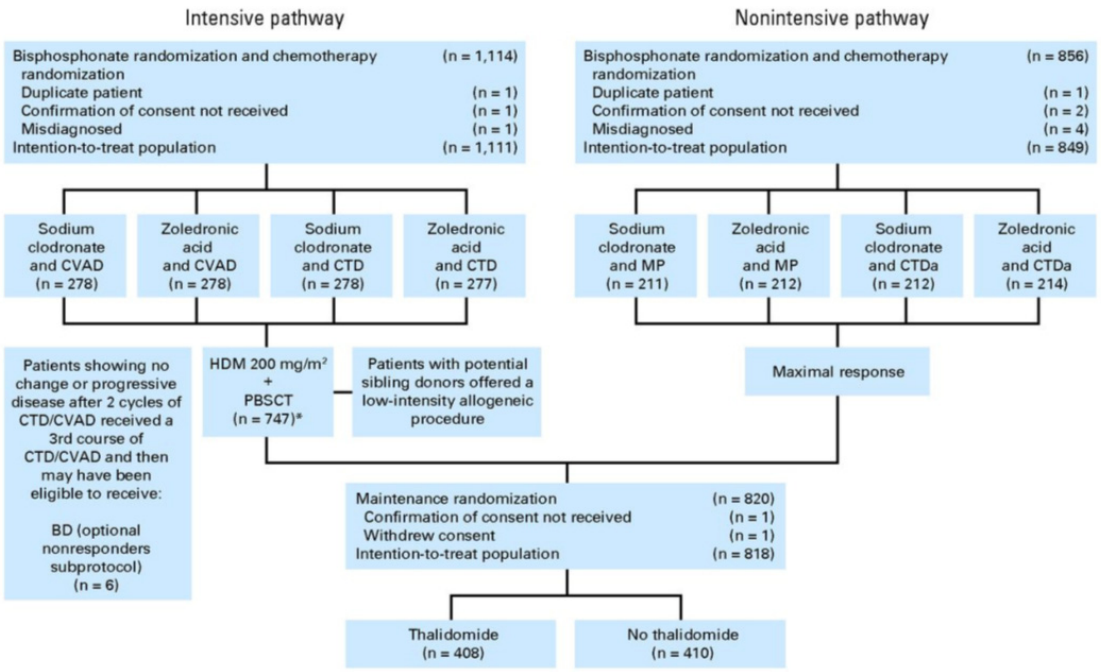

SUPPLEMENTARY REFERENCES

1. Jackson GH, Davies FE, Pawlyn C, Cairns DA, Striha A, Collett C*, et al.* Lenalidomide maintenance versus observation for patients with newly diagnosed multiple myeloma (Myeloma XI): a multicentre, open-label, randomised, phase 3 trial. *The lancet oncology* 2019 Jan; **20**(1)**:** 57-73.

2. Morgan GJ, Davies FE, Gregory WM, Bell SE, Szubert AJ, Cook G*, et al.* Long-term follow-up of MRC Myeloma IX trial: Survival outcomes with bisphosphonate and thalidomide treatment. *Clinical cancer research : an official journal of the American Association for Cancer Research* 2013 Nov 1; **19**(21)**:** 6030-6038.

3. Shah V, Sherborne AL, Walker BA, Johnson DC, Boyle EM, Ellis S*, et al.* Prediction of outcome in newly diagnosed myeloma: a meta-analysis of the molecular profiles of 1905 trial patients. *Leukemia : official journal of the Leukemia Society of America, Leukemia Research Fund, UK* 2018 Jan; **32**(1)**:** 102-110.

4. Kuiper R, Broyl A, de Knegt Y, van Vliet MH, van Beers EH, van der Holt B*, et al.* A gene expression signature for high-risk multiple myeloma. *Leukemia : official journal of the Leukemia Society of America, Leukemia Research Fund, UK* 2012 Nov; **26**(11)**:** 2406-2413.

5. Shaughnessy JD, Jr., Qu P, Usmani S, Heuck CJ, Zhang Q, Zhou Y*, et al.* Pharmacogenomics of bortezomib test-dosing identifies hyperexpression of proteasome genes, especially PSMD4, as novel high-risk feature in myeloma treated with Total Therapy 3. *Blood* 2011 Sep 29; **118**(13)**:** 3512-3524.

6. Shaughnessy JD, Zhan F, Burington BE, Huang Y, Colla S, Hanamura I*, et al.* A validated gene expression model of high-risk multiple myeloma is defined by deregulated expression of genes mapping to chromosome 1. *Blood* 2007 Mar 15; **109**(6)**:** 2276-2284.

7. Dickens NJ, Walker BA, Leone PE, Johnson DC, Brito JL, Zeisig A*, et al.* Homozygous deletion mapping in myeloma samples identifies genes and an expression signature relevant to pathogenesis and outcome. *Clinical cancer research : an official journal of the American Association for Cancer Research* 2010 Mar 15; **16**(6)**:** 1856-1864.

8. Decaux O, Lode L, Magrangeas F, Charbonnel C, Gouraud W, Jezequel P*, et al.* Prediction of survival in multiple myeloma based on gene expression profiles reveals cell cycle and chromosomal instability signatures in high-risk patients

and hyperdiploid signatures in low-risk patients: a study of the Intergroupe Francophone du Myelome. *Journal of clinical oncology : official journal of the American Society of Clinical Oncology* 2008 Oct 10; **26**(29)**:** 4798-4805.

9. Moreaux J, Klein B, Bataille R, Descamps G, Maiga S, Hose D*, et al.* A high-risk signature for patients with multiple myeloma established from the molecular classification of human myeloma cell lines. *Haematologica* 2011 Apr; **96**(4)**:** 574-582.

DATA-SHARING STATEMENT

Gene expression data has been deposited under EGA accession EGAS00001004183; requests will be reviewed by the data access committee. Only non-commercial requests will be considered. De-identified participant data will be made available when all primary and secondary endpoints have been met. Requests for trial data and supporting material (data dictionary, protocol, and statistical-analysis plan) will be reviewed by the trial-management group in the first instance. Only requests that have a methodologically sound proposal and whose proposed use of the data has been approved by the independent trial steering committee will be considered. Proposals should be directed to the corresponding author in the first instance; to gain access, data requestors will need to sign a data access agreement.

SUPPLEMENTARY TABLES

Supplementary Table 1. Baseline characteristics of representative transplant-eligible patients included in this analysis (left column), those not included (middle column) and all transplant-eligible trial patients (right column) from NCRI Myeloma XI.

	**Current study (n=329)**	**Others (n=1713)**	**Total (n=2042)**
Gender (Male)	197 (59.9%)	1024 (59.8%)	1221 (59.8%)
Age at randomization >65 years	81 (24.6%)	435 (25.4%)	516 (25.3%)
**WHO performance status**			
0	135 (41.0%)	729 (42.6%)	864 (42.3%)
1	124 (37.7%)	608 (35.5%)	732 (35.8%)
2	38 (11.6%)	219 (12.8%)	257 (12.6%)
3	16 (4.9%)	62 (3.6%)	78 (3.8%)
4	3 (0.9%)	7 (0.4%)	10 (0.5%)
**Haemoglobin (g/L)** Mean (SD)	106.6 (19.01)	110.0 (20.41)	109.5 (20.23)
**Platelets (*10/L)** Mean (SD)	248.3 (98.90)	252.4 (101.78)	251.7 (101.30)
**ß2 microglobulin (mg/L)** Median (Range)	3.8 (1.5, 41.0)	3.6 (0.6, 81.2)	3.6 (0.6, 81.2)
**Calcium (mmol/l)** Median (Range)	2.4 (1.8, 3.8)	2.4 (1.3, 4.6)	2.4 (1.3, 4.6)
**Serum creatinine (µmol/L)**			
Mean (SD)	102.8 (58.97)	99.1 (62.45)	99.7 (61.91)
Median (Range)	87.0 (37.0, 405.0)	83.0 (28.0, 897.0)	84.0 (28.0, 897.0)
**Paraprotein type**			
IgG	196 (59.6%)	1042 (60.8%)	1238 (60.6%)
IgA	83 (25.2%)	409 (23.9%)	492 (24.1%)
**Light chain type**			
Lambda	97 (29.5%)	587 (34.3%)	684 (33.5%)
Kappa	231 (70.2%)	1106 (64.6%)	1337 (65.5%)
**Serum Albumin (g/L)** Mean (SD)	34.6 (7.13)	35.6 (6.88)	35.4 (6.93)
**Lactate dehydrogenase (IU/L)** Median (Range)	264.0 (<lower detection limit, 1042.0)	268.0 (<lower detection limit, 3550.0)	267.0 (<lower detection limit, 3550.0)
**C-Reactive protein (mg/L)** >=5	168 (51.1%)	880 (51.4%)	1048 (51.3%)

Supplementary Table 2. Baseline demographics, clinical and laboratory characteristics of patients with tumors classified as SKY92 standard-risk or high-risk.

	**SKY92 Standard-Risk (N=248)**	**SKY92 High-risk (N=81)**	***P***
**Demographics**			
Age ≥ 65	58 (23.4)	23 (28.4)	0.447
Gender (Male)	152 (61.3)	45 (55.6)	0.433
**Clinical Characteristics**			
WHO Performance Score ≥ 2 (%)	35 (14.9)	22 (27.2)	0.021
ISS (%)			0.003
I	83 (33.6)	15 (18.5)	
II	112 (45.3)	35 (43.2)	
III	52 (21.1)	31 (38.3)	
β2M mg/L (median [IQR])	3.60 [2.70, 4.95]	4.50 [3.30, 6.90]	0.002
Albumin <35g/dL (%)	86 (34.7)	38 (46.9)	0.07
CRP (median [IQR])	3.10 [1.20, 9.50]	5.20 [1.05, 13.50]	0.247
Serum Creatinine (median [IQR])	85.50 [71.00, 103.25]	91.00 [74.00, 118.00]	0.111
Urea (median [IQR])	5.50 [4.60, 7.50]	5.90 [4.60, 8.60]	0.108
Haemoglobin g/L (mean (sd))	108.95 (18.21)	99.53 (19.75)	<0.001
Platelets (mean (sd))	261.95 (93.19)	206.68 (104.66)	<0.001
Lactate dehydrogenase units/L (mean (sd))	269.18 (135.26)	363.19 (222.35)	<0.001
Calcium mmol/L > 2.6 (%)	39 (15.8)	23 (28.4)	0.019
Bone Disease (%)	163 (70.9)	47 (63.5)	0.295
**Genetic Characteristics**			
Adverse translocation (%)	45 (18.1)	35 (43.2)	<0.001
t(4;14) (%)	40 (16.1)	28 (34.6)	0.001
t(14;16) or t(14;20) (%)	5 (2.0)	7 (8.6)	0.016
t(11;14) (%)	49 (19.8)	7 (8.6)	0.032
Hyperdiploid (%)	134 (54.0)	26 (32.1)	0.001
Gain(1q) [CKS1B] (%)	70 (28.2)	50 (61.7)	<0.001
Del(1p) [CDKN2C] (%)	17 (6.9)	19 (23.5)	<0.001
Del(17p) [TP53] (%)	21 (8.5)	13 (16.0)	0.083
Del(13q) (%)	89 (35.9)	54 (66.7)	<0.001

Supplementary Table 3. Cox-based univariate and multivariate analysis for induction randomization arms cyclophosphamide, thalidomide and dexamethasone (CTD) and cyclophosphamide, lenalidomide and dexamethasone (CRD).

CTD Induction

Univariate Analysis

Progression Free Survival			
	N	HR (95% CI)	Wald *P*
SKY92 high-risk	144	2.67 (1.78-4)	1.81E-06
Hyperdiploid	144	0.62 (0.42-0.91)	0.0142
Adverse translocation	144	2.39 (1.59-3.58)	2.49E-05
Del(1p) [CDKN2C]	144	1.74 (0.97-3.12)	0.0637
Del(17p) [TP53]	144	1.83 (1.06-3.17)	0.0308
Gain(1q)	144	1.55 (1.06-2.27)	0.0227
ISS	144	1.37 (1.08-1.75)	0.00987
Age	144	1.02 (1-1.05)	0.0612

Multivariate Analysis

Progression Free Survival		
	HR (95% CI)	Wald *P*
SKY92 high-risk	2.32 (1.41-3.84)	0.001
Hyperdiploid	0.81 (0.54-1.22)	0.317
Adverse translocation	1.94 (1.21-3.12)	0.00577
Del(1p) [CDKN2C]	0.69 (0.32-1.46)	0.329
Del(17p) [TP53]	1.68 (0.86-3.29)	0.132
Gain(1q)	0.89 (0.55-1.44)	0.632
ISS	1.1 (0.85-1.43)	0.477
Age	1.03 (1.01-1.06)	0.0197

Overall Survival			
	N	HR (95% CI)	Wald *P*
SKY92 high-risk	144	3.51 (2.14-5.76)	6.71E-07
Hyperdiploid	144	0.81 (0.49-1.33)	0.404
Adverse translocation	144	2.1 (1.27-3.48)	0.00374
Del(1p) [CDKN2C]	144	2.69 (1.4-5.17)	0.00303
Del(17p) [TP53]	144	2.85 (1.51-5.38)	0.00125
Gain(1q)	144	2.49 (1.52-4.09)	0.000319
ISS	144	1.45 (1.04-2.02)	0.0273
Age	144	1.02 (0.99-1.06)	0.178

Overall Survival		
	HR (95% CI)	Wald *P*
SKY92 high-risk	2.64 (1.42-4.91)	0.00213
Hyperdiploid	1.07 (0.63-1.83)	0.792
Adverse translocation	1.52 (0.82-2.81)	0.182
Del(1p) [CDKN2C]	0.97 (0.43-2.16)	0.936
Del(17p) [TP53]	2.55 (1.18-5.5)	0.0174
Gain(1q)	1.27 (0.68-2.37)	0.445
ISS	1.1 (0.76-1.61)	0.607
Age	1.02 (0.98-1.06)	0.323

CRD Induction

Progression Free Survival			
	N	HR (95% CI)	Wald *P*
SKY92 high-risk	185	2.45 (1.63-3.68)	1.67E-05
Hyperdiploid	185	0.87 (0.61-1.25)	0.454
Adverse translocation	185	1.69 (1.1-2.58)	0.0156
Del(1p) [CDKN2C]	185	1.38 (0.81-2.33)	0.237
Del(17p) [TP53]	185	1.44 (0.81-2.57)	0.215
Gain(1q)	185	1.3 (0.89-1.89)	0.174
ISS	184	1.32 (1.03-1.69)	0.0256
Age	185	1.05 (1.02-1.08)	0.00069

Progression Free Survival		
	HR (95% CI)	Wald *P*
SKY92 high-risk	2.01 (1.3-3.13)	0.00187
Hyperdiploid	1.1 (0.72-1.67)	0.668
Adverse translocation	2 (1.22-3.28)	0.00623
Del(1p) [CDKN2C]	1.24 (0.7-2.18)	0.46
Del(17p) [TP53]	1.28 (0.72-2.3)	0.403
Gain(1q)	0.86 (0.57-1.31)	0.489
ISS	1.15 (0.89-1.5)	0.284
Age	1.04 (1.01-1.07)	0.0038

Overall Survival			
	N	HR (95% CI)	Wald *P*
SKY92 high-risk	185	4.22 (2.43-7.32)	2.96E-07
Hyperdiploid	185	0.46 (0.26-0.8)	0.00643
Adverse translocation	185	2.9 (1.64-5.11)	0.000234
Del(1p) [CDKN2C]	185	2.29 (1.17-4.48)	0.015
Del(17p) [TP53]	185	3.04 (1.47-6.28)	0.00262
Gain(1q)	185	2.12 (1.23-3.65)	0.00678
ISS	184	1.36 (0.94-1.97)	0.0994
Age	185	1.03 (0.99-1.07)	0.154

Overall Survival		
	HR (95% CI)	Wald *P*
SKY92 high-risk	3.19 (1.76-5.8)	0.000139
Hyperdiploid	0.84 (0.41-1.69)	0.619
Adverse translocation	2.69 (1.32-5.46)	0.00626
Del(1p) [CDKN2C]	1.83 (0.87-3.87)	0.113
Del(17p) [TP53]	2.51 (1.17-5.37)	0.0176
Gain(1q)	1.38 (0.76-2.52)	0.293
ISS	1.11 (0.75-1.65)	0.601
Age	1.01 (0.97-1.05)	0.624

Supplementary Table 4. Cox-based univariate and multivariate analysis landmarked from time point of HDMEL and ASCT for PFS and OS.

			**Univariate Analysis**				**Multivariate Analysis**	
Progression Free Survival						Progression Free Survival		
	N	n events	HR (95% CI)	Wald *P*			HR (95% CI)	Wald *P*
SKY92 high-risk	205	123	2.95 (2-4.37)	5.99E-08		SKY92 high-risk	2.53 (1.61 - 3.97)	5.22E-05
Adverse translocation	205	123	2.19 (1.48-3.24)	8.82E-05		Adverse translocation	2.25 (1.44 - 3.5)	0.000341
Del(1p) [CDKN2C]	205	123	1.72 (1.02-2.92)	0.0434		Del(1p) [CDKN2C]	1.07 (0.59 - 1.95)	0.826
Del(17p) [TP53]	205	123	2.12 (1.27-3.55)	0.00412		Del(17p) [TP53]	1.63 (0.95 - 2.8)	0.0765
Gain(1q)	205	123	1.62 (1.13-2.32)	0.00916		Gain(1q)	0.97 (0.63 - 1.5)	0.902
Hyperdiploid	205	123	0.84 (0.59-1.19)	0.326		Hyperdiploid	1.12 (0.76 - 1.66)	0.567
Age	205	123	1.03 (1.01-1.06)	0.00781		Age	1.04 (1.01 - 1.06)	0.00676
ISS	204	123	1.2 (0.94-1.52)	0.136		ISS	0.97 (0.76 - 1.25)	0.825
Double-hit	205	123	3.17 (2.1-4.8)	4.47E-08				
Overall Survival						Overall Survival		
	N	n events	HR (95% CI)	Wald *P*			HR (95% CI)	Wald *P*
SKY92 high-risk	205	56	3.93 (2.31-6.69)	4.76E-07		SKY92 high-risk	2.92 (1.57 - 5.42)	0.000675
Adverse translocation	205	56	2.26 (1.31-3.9)	0.00357		Adverse translocation	2.01 (1.05 - 3.82)	0.0346
Del(1p) [CDKN2C]	205	56	3.5 (1.83-6.71)	0.000161		Del(1p) [CDKN2C]	1.55 (0.73 - 3.31)	0.254
Del(17p) [TP53]	205	56	4.76 (2.58-8.77)	5.69E-07		Del(17p) [TP53]	3.94 (2 - 7.76)	7.13E-05
Gain(1q)	205	56	2.91 (1.71-4.94)	8.16E-05		Gain(1q)	1.67 (0.9 - 3.12)	0.105
Hyperdiploid	205	56	0.77 (0.46-1.3)	0.334		Hyperdiploid	1.28 (0.69 - 2.37)	0.438
Age	205	56	1.02 (0.99-1.06)	0.256		Age	1 (0.96 - 1.04)	0.989
ISS	204	56	1.19 (0.83-1.7)	0.337		ISS	0.91 (0.62 - 1.34)	0.637
Double-hit	205	56	5.03 (2.93-8.64)	4.98E-09				

Supplementary Table 5. Cox-based univariate and multivariate analysis landmarked from time point of maintenance randomization to lenalidomide or observation for PFS and OS.

			Univariate Analysis	
Progression Free Survival				
	N	n events	HR (95% CI)	Wald *P*
SKY92 high-risk	149	87	3.34 (2.1-5.31)	3.55E-07
Adverse translocation	149	87	2.09 (1.3-3.36)	0.00234
Del(1p) [CDKN2C]	149	87	1.77 (0.88-3.55)	0.108
Del(17p) [TP53]	149	87	2.05 (1.13-3.71)	0.0176
Gain(1q)	149	87	1.73 (1.12-2.66)	0.0128
Hyperdiploid	149	87	0.84 (0.55-1.28)	0.427
Age	149	87	1.03 (1-1.07)	0.0258
ISS	148	87	1.17 (0.89-1.54)	0.257
Maintenance Randomization Len vs Observation	149	87	0.42 (0.27-0.65)	0.000116

	Multivariate Analysis	
Progression Free Survival		
	HR (95% CI)	Wald *P*
SKY92 high-risk	2.61 (1.54-4.41)	0.000343
Adverse translocation	2.01 (1.15-3.51)	0.0142
Del(1p) [CDKN2C]	1.35 (0.6-3.05)	0.475
Del(17p) [TP53]	1.94 (1.02-3.72)	0.0444
Gain(1q)	1.31 (0.77-2.21)	0.32
Hyperdiploid	1.09 (0.67-1.76)	0.739
Age	1.03 (1-1.06)	0.0811
ISS	0.92 (0.69-1.22)	0.563
Maintenance Randomization Len vs Observation	0.34 (0.21-0.54)	6.74E-06

Overall Survival				
	N	n events	HR (95% CI)	Wald *P*
SKY92 high-risk	149	38	3.92 (2.04-7.54)	4.23E-05
Adverse translocation	149	38	2.18 (1.11-4.26)	0.0231
Del(1p) [CDKN2C]	149	38	3.2 (1.39-7.36)	0.00607
Del(17p) [TP53]	149	38	4.29 (2.08-8.87)	8.33E-05
Gain(1q)	149	38	3.18 (1.67-6.07)	0.00045
Hyperdiploid	149	38	0.85 (0.45-1.62)	0.63
Age	149	38	1.03 (0.98-1.07)	0.245
ISS	148	38	1.09 (0.72-1.66)	0.677
Maintenance Randomization Len vs Observation	149	38	0.88 (0.47-1.67)	0.707

Overall Survival		
	HR (95% CI)	Wald *P*
SKY92 high-risk	3.13 (1.46-6.71)	0.00324
Adverse translocation	1.84 (0.81-4.16)	0.144
Del(1p) [CDKN2C]	1.26 (0.48-3.35)	0.637
Del(17p) [TP53]	4.4 (1.94-9.99)	0.000401
Gain(1q)	2.4 (1.11-5.17)	0.0257
Hyperdiploid	1.34 (0.61-2.94)	0.466
Age	1 (0.95-1.05)	0.968
ISS	0.82 (0.53-1.28)	0.381
Maintenance Randomization Len vs Observation	0.56 (0.28-1.14)	0.108

Supplementary Table 6. Cox-based univariate and multivariate analysis from trial entry for PFS and OS with UAMS GEP70 risk status.

Progression Free Survival

			**Univariate analysis**	
	N	events	HR (95% CI)	Wald *P*
UAMS70 High-risk	329	232	1.86 (1.34-2.59)	0.000234
Del(1p) [CDKN2C]	329	232	1.47 (1-2.18)	0.0514
Del(17p) [TP53]	329	232	1.63 (1.09-2.42)	0.016
Gain(1q)	329	232	1.44 (1.11-1.88)	0.00634
Adverse translocation	329	232	2.04 (1.53-2.72)	1.12E-06
Hyperdiploid	329	232	0.74 (0.57-0.95)	0.0198
ISS	328	232	1.33 (1.12-1.58)	0.0012
Age	329	232	1.04 (1.02-1.06)	0.000115
Induction Randomization	329	232	1.31 (1.01-1.69)	0.0417

	**Multivariate analysis**	
	HR (95% CI)	Wald *P*
UAMS70 High-risk	1.37 (0.91 - 2.04)	0.129
Del(1p) [CDKN2C]	1.04 (0.66 - 1.64)	0.867
Del(17p) [TP53]	1.35 (0.89 - 2.05)	0.163
Gain(1q)	1.02 (0.75 - 1.38)	0.891
Adverse translocation	2.01 (1.45 - 2.79)	0.00003
Hyperdiploid	0.93 (0.7 - 1.23)	0.605
ISS	1.17 (0.97 - 1.4)	0.106
Age	1.04 (1.02 - 1.06)	0.000143
Induction Randomization	1.21 (0.93 - 1.57)	0.152

**Overall Survival**			**Univariate analysis**	
	N	events	HR (95% CI)	Wald *P*
UAMS70 High-risk	329	117	3.79 (2.55-5.65)	5.51E-11
Del(1p) [CDKN2C]	329	117	2.38 (1.49-3.79)	0.000271
Del(17p) [TP53]	329	117	3.02 (1.87-4.87)	5.76E-06
Gain(1q)	329	117	2.39 (1.66-3.44)	2.98E-06
Adverse translocation	329	117	2.5 (1.72-3.64)	1.67E-06
Hyperdiploid	329	117	0.6 (0.42-0.87)	0.00717
ISS	328	117	1.38 (1.08-1.76)	0.0101
Age	329	117	1.03 (1-1.05)	0.033
Induction Randomization	329	117	1.6 (1.11-2.31)	0.0113

	**Multivariate analysis**	
	HR (95% CI)	Wald *P*
UAMS70 High-risk	2.54 (1.56 - 4.13)	0.000175
Del(1p) [CDKN2C]	1.19 (0.68 - 2.08)	0.545
Del(17p) [TP53]	2.22 (1.32 - 3.72)	0.00253
Gain(1q)	1.39 (0.91 - 2.12)	0.125
Adverse translocation	2.11 (1.35 - 3.28)	0.000951
Hyperdiploid	0.95 (0.62 - 1.44)	0.806
ISS	1.1 (0.84 - 1.43)	0.505
Age	1.02 (1 - 1.05)	0.0972
Induction Randomization	1.39 (0.96 - 2.02)	0.079

Supplementary Table 7. Cox-based univariate and multivariate survival analysis of prognostic association of molecular or clinical variables for 161 patients without MPCA risk markers.

			**Univariate Analysis**	
Progression Free Survival				
	N	events	HR (95% CI)	Wald *P*
SKY92 high-risk	161	104	3.91 (2.34-6.54)	1.85E-07
Del(1p) [CDKN2C]	161	104	3.48 (1.73-7)	0.000464
Hyperdiploidy	161	104	1.1 (0.73-1.65)	0.66
Age	161	104	1.05 (1.02-1.08)	0.00157
Induction CTD vs CRD	161	104	1.02 (0.69-1.5)	0.933
ISS	161	104	1.23 (0.96-1.58)	0.0991

	**Multivariate Analysis**	
Progression Free Survival		
	HR (95% CI)	Wald *P*
SKY92 high-risk	3.18 (1.86-5.46)	2.57E-05
Del(1p) [CDKN2C]	2.26 (1.07-4.78)	0.0335
Hyperdiploidy	1.14 (0.75-1.73)	0.529
Age	1.04 (1.01-1.06)	0.0162
Induction CTD vs CRD	1.05 (0.69-1.58)	0.834
ISS	1.09 (0.85-1.41)	0.496

Overall Survival				
	N	events	HR (95% CI)	Wald *P*
SKY92 high-risk	161	35	3.51 (1.68-7.31)	0.000809
Del(1p) [CDKN2C]	161	35	3.36 (1.3-8.68)	0.0124
Hyperdiploidy	161	35	0.95 (0.48-1.89)	0.884
Age	161	35	1.02 (0.97-1.06)	0.448
Induction CTD vs CRD	161	35	1.59 (0.82-3.1)	0.172
ISS	161	35	1.5 (0.97-2.32)	0.0656

Overall Survival		
	HR (95% CI)	Wald *P*
SKY92 high-risk	2.42 (1.04-5.67)	0.0412
Del(1p) [CDKN2C]	2.33 (0.77-7.06)	0.134
Hyperdiploidy	1.03 (0.5-2.13)	0.927
Age	1 (0.95-1.05)	0.951
Induction CTD vs CRD	1.78 (0.87-3.62)	0.113
ISS	1.37 (0.87-2.15)	0.177

Supplementary Table 8. Cox based univariate and multivariate analysis of PFS and OS from time point of a) induction randomization b) maintenance randomization.

**a) From induction randomization**								
			**Univariate Analysis**				**Multivariate Analysis**	
Progression Free Survival						Progression Free Survival		
	N	events	HR (95% CI)	Wald *P*			HR (95% CI)	Wald *P*
SKY92 high-risk	329	232	2.6 (1.96-3.45)	4.08E-11		SKY92 high-risk	2.02 (1.49 - 2.75)	6.76E-06
Double-Hit	329	232	2.36 (1.73-3.22)	5.43E-08		Double-Hit	1.64 (1.16 - 2.31)	0.00499
Hyperdiploid	329	232	0.74 (0.57-0.95)	0.0198		Hyperdiploid	0.85 (0.65 - 1.12)	0.248
ISS	328	232	1.33 (1.12-1.58)	0.0012		ISS	1.15 (0.96 - 1.38)	0.12
Age	329	232	1.04 (1.02-1.06)	0.000115		Age	1.03 (1.01 - 1.05)	0.000866
Induction CTD vs CRD	329	232	1.31 (1.01-1.69)	0.0417		Induction CTD vs CRD	1.18 (0.91 - 1.53)	0.223
Overall Survival						Overall Survival		
			Univariate Analysis				Multivariate Analysis	
	N	events	HR (95% CI)	Wald *P*			HR (95% CI)	Wald *P*
SKY92 high-risk	329	117	3.94 (2.73-5.69)	2.54E-13		SKY92 high-risk	2.85 (1.91 - 4.24)	2.63E-07
Double-hit	329	117	3.72 (2.54-5.47)	1.98E-11		Double-hit	2.3 (1.49 - 3.57)	0.000193
Hyperdiploid	329	117	0.6 (0.42-0.87)	0.00717		Hyperdiploid	0.83 (0.56 - 1.24)	0.373
ISS	328	117	1.38 (1.08-1.76)	0.0101		ISS	1.15 (0.88 - 1.49)	0.315
Age	329	117	1.03 (1-1.05)	0.033		Age	1.02 (0.99 - 1.04)	0.263
Induction CTD vs CRD	329	117	1.6 (1.11-2.31)	0.0113		Induction CTD vs CRD	1.27 (0.87 - 1.85)	0.218

**b) Landmarked from maintenance randomization**								
Progression Free Survival						Progression Free Survival		
			Univariate Analysis				Multivariate Analysis	
	N	events	HR (95% CI)	Wald *P*			HR (95% CI)	Wald *P*
SKY92 high-risk	149	87	3.34 (2.1-5.31)	3.55E-07		SKY92 high-risk	2.71 (1.64 - 4.48)	9.64E-05
Double-Hit	149	87	3.15 (1.93-5.13)	4.28E-06		Double-Hit	2.78 (1.61 - 4.8)	0.000246
Hyperdiploid	149	87	0.84 (0.55-1.28)	0.427		Hyperdiploid	1.02 (0.65 - 1.62)	0.918
ISS	148	87	1.17 (0.89-1.54)	0.257		ISS	0.92 (0.7 - 1.22)	0.571
Age	149	87	1.03 (1-1.07)	0.0258		Age	1.03 (0.99 - 1.06)	0.107
Maintenance Len vs Obs	149	87	0.42 (0.27-0.65)	0.000116		Maintenance Len vs Obs	0.39 (0.25 - 0.6)	2.73E-05
Overall Survival						Overall Survival		
			Univariate Analysis				Multivariate Analysis	
	N	events	HR (95% CI)	Wald *P*			HR (95% CI)	Wald *P*
SKY92 high-risk	149	38	3.92 (2.04-7.54)	4.23E-05		SKY92 high-risk	3.31 (1.64 - 6.69)	0.000866
Double-hit	149	38	4.74 (2.46-9.14)	3.27E-06		Double-Hit	4.58 (2.15 - 9.79)	8.36E-05
Hyperdiploid	149	38	0.85 (0.45-1.62)	0.63		Hyperdiploid	1.25 (0.61 - 2.56)	0.544
ISS	148	38	1.09 (0.72-1.66)	0.677		ISS	0.79 (0.52 - 1.23)	0.298
Age	149	38	1.03 (0.98-1.07)	0.245		Age	1.01 (0.96 - 1.05)	0.776
Maintenance Len vs Obs	149	38	0.88 (0.47-1.67)	0.707		Maintenance Len vs Obs	0.77 (0.4 - 1.5)	0.449

Supplementary Table 9. Cox based multivariable analysis of PFS and OS from time point of a) Induction randomization and b) transplant consolidation of cytogenetic and SKY92 risk scoring strata.

a) **Multivariate analysis**

Progression Free Survival

	HR (95% CI)	Wald *P*
No high-risk	1	n/a
1 cytogenetic high-risk lesion	1.32 (0.93 - 1.86)	0.118
Double-hit OR SKY92 high-risk	2.34 (1.68 - 3.26)	4.44E-07
Double-hit AND SKY92 high-risk	4.5 (2.96 - 6.86)	2.25E-12

Overall Survival

	HR (95% CI)	Wald *P*
No high-risk	1	n/a
1 cytogenetic high-risk lesion	1.88 (1.08 - 3.28)	0.0255
Double-hit OR SKY92 high-risk	3.8 (2.3 - 6.29)	2.00E-07
Double-hit AND SKY92 high-risk	10.98 (6.32 - 19.09)	2.01E-17

b) **Multivariate Analysis**

Progression Free Survival

	HR (95% CI)	Wald *P*
No high-risk	1	n/a
1 cytogenetic high-risk lesion	1.12 (0.68 - 1.83)	0.653
Double-hit OR SKY92 high-risk	2.61 (1.68 - 4.08)	2.31E-05
Double-hit AND SKY92 high-risk	6.2 (3.49 - 11.02)	4.59E-10

Overall Survival

	HR (95% CI)	Wald *P*
No high-risk	1	n/a
1 cytogenetic high-risk lesion	1.65 (0.74 - 3.69)	0.221
Double-hit OR SKY92 high-risk	3.59 (1.76 - 7.34)	0.000454
Double-hit AND SKY92 high-risk	14.47 (6.69 - 31.26)	1.08E-11

Supplementary Table 10. Cox based multivariable analysis of PFS and OS from induction randomization including LDH, other clinical and molecular risk markers.

Multivariate Analysis

Progression Free Survival

	HR (95% CI)	Wald *P*
SKY92 high-risk	1.9 (1.34 - 2.69)	0.000282
Double-hit	1.84 (1.28 - 2.65)	0.000953
ISS	1.09 (0.9 - 1.33)	0.373
Age	1.03 (1.01 - 1.05)	0.00221
Induction CTD vs CRD	1.13 (0.85 - 1.5)	0.404
Lactate dehydrogenase units/L	1 (1 - 1)	0.805

Overall Survival

	HR (95% CI)	Wald *P*
SKY92 high-risk	3.01 (1.93 - 4.7)	1.30E-06
Double-hit	2.24 (1.41 - 3.57)	0.000631
ISS	1.12 (0.84 - 1.49)	0.449
Age	1.02 (0.99 - 1.05)	0.156
Induction CTD vs CRD	1.25 (0.83 - 1.9)	0.284
Lactate dehydrogenase units/L	1 (1 - 1)	0.283

SUPPLEMENTARY FIGURES

Supplementary Figure 1

Kaplan-Meier plot of the analyzed representative NCRI Myeloma XI trial patients (n=329) in context of SKY92 risk profiling results for A) PFS, B) OS from induction randomization. Log-rank *P*-values displayed.

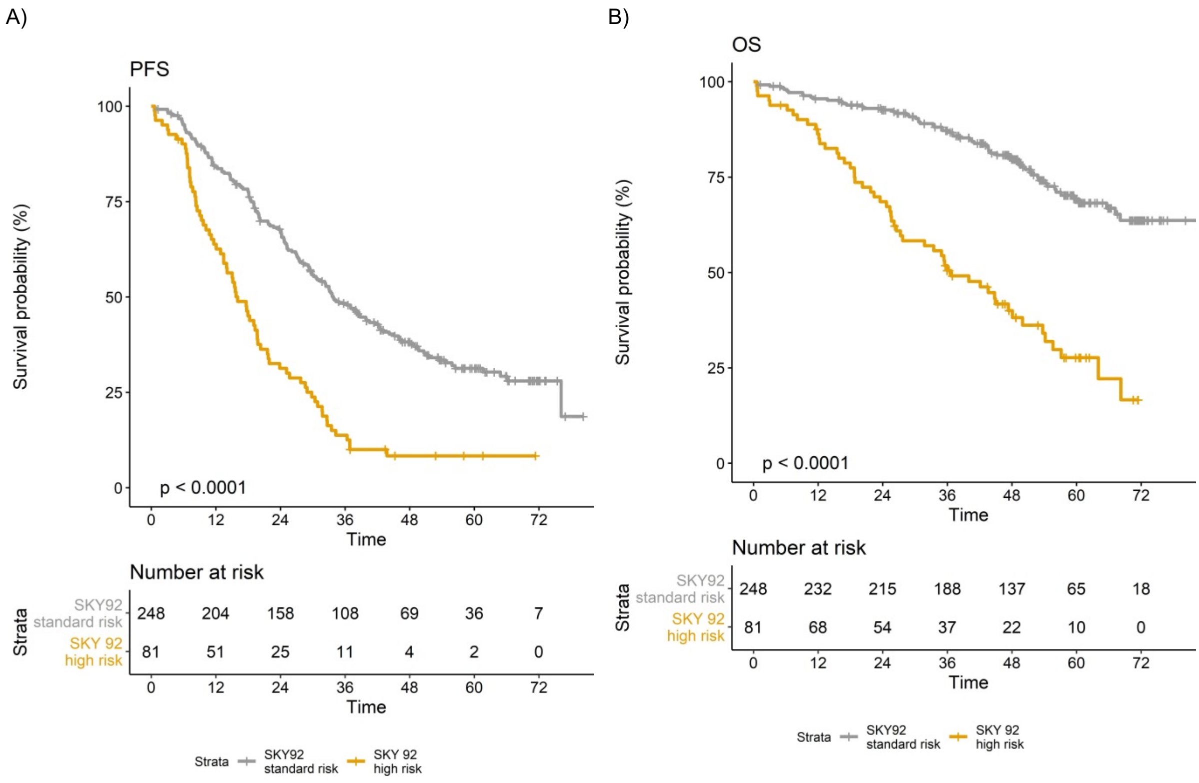

Supplementary Figure 2

Kaplan-Meier plots for groups defined by presence or absence of SKY92 GEP risk status in patients randomised (ITT) to induction with CTD (A, B) or CRD (C, D).

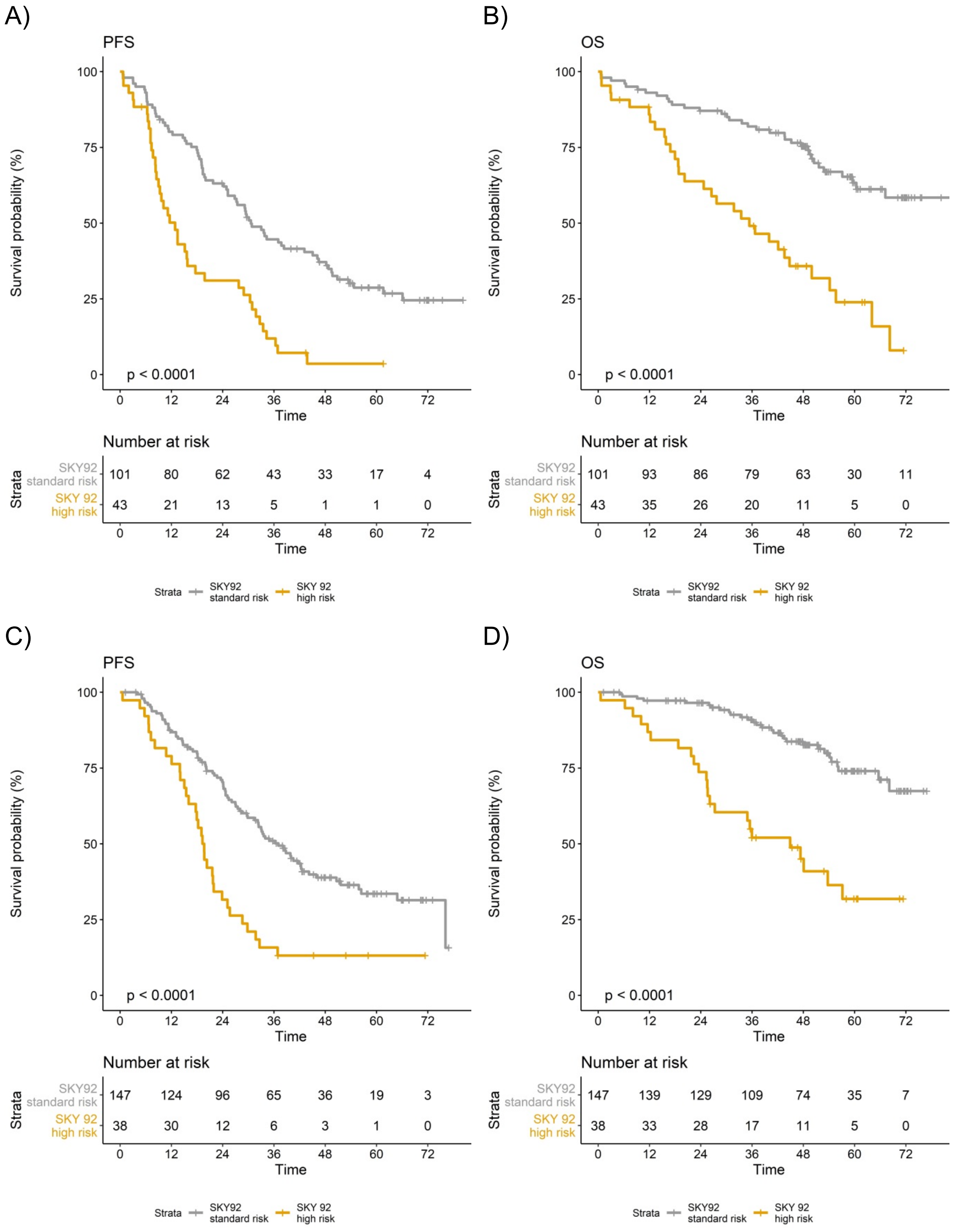

Supplementary Figure 3

Kaplan-Meier plots for PFS and OS for double-hit (A,B,E,F,I,J) or SKY92 (C,D,G,H,K,L) high-risk absent vs landmarked from induction randomization (A,B,C,D), post high dose melphalan and autologous stem cell transplant (E,F,G,H) and randomization to lenalidomide maintenance (subset to only patients randomized to lenalidomide monotherapy)(I,J,K,L).

Double-hit vs other **SKY92 High-Risk vs other**

Landmarked post induction randomisation

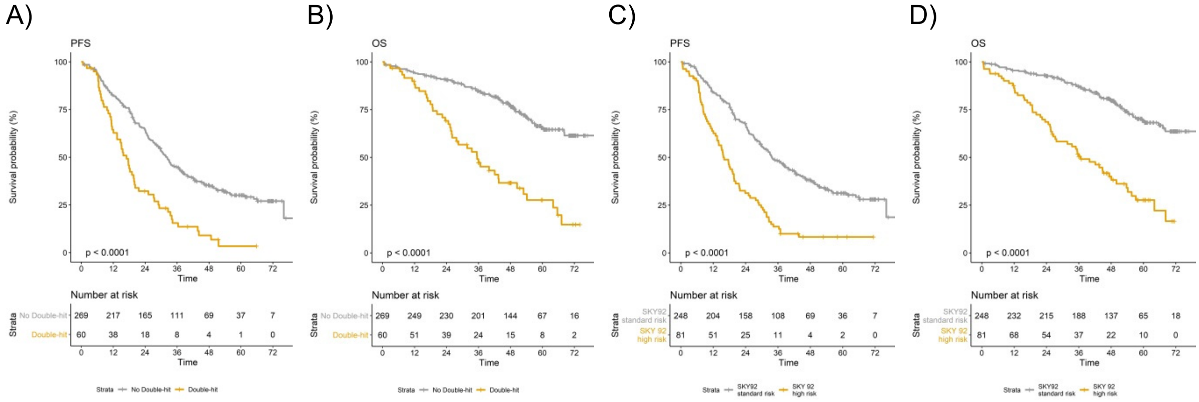

Landmarked post transplantation

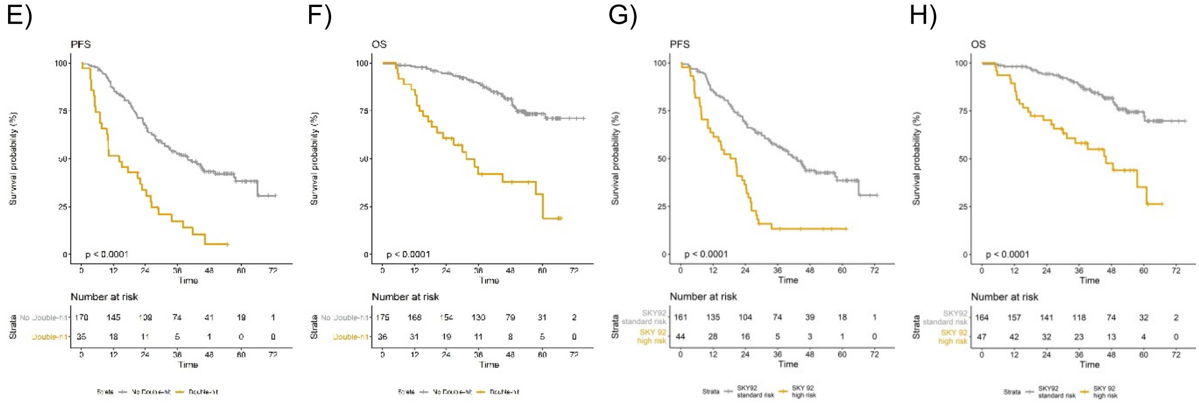

Landmarked from lenalidomide randomization (subset to patients who were randomized to receive lenalidomide)

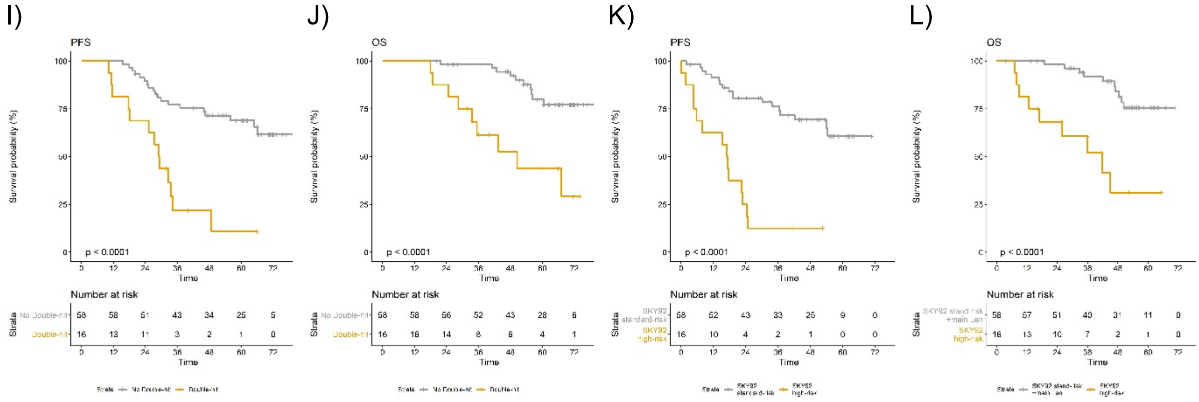

Supplementary Figure 4

Kaplan-Meier plots of subset of 161 patients with no chromosomal high-risk markers in context of presence or absence of SKY92 high-risk status A) PFS B) OS.

A)

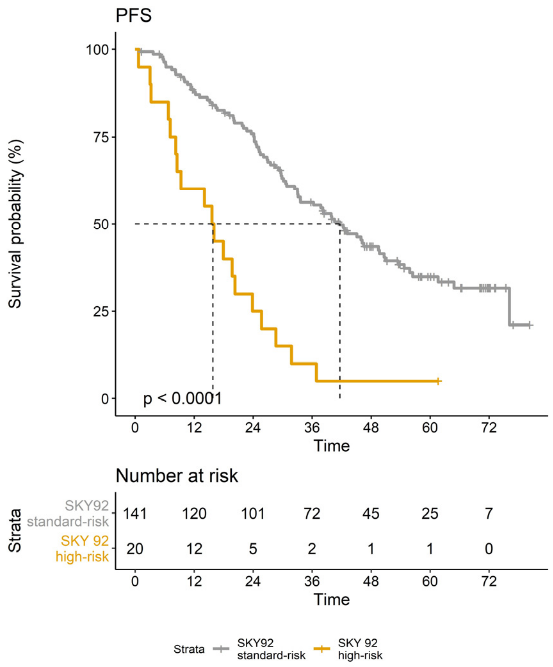

B)

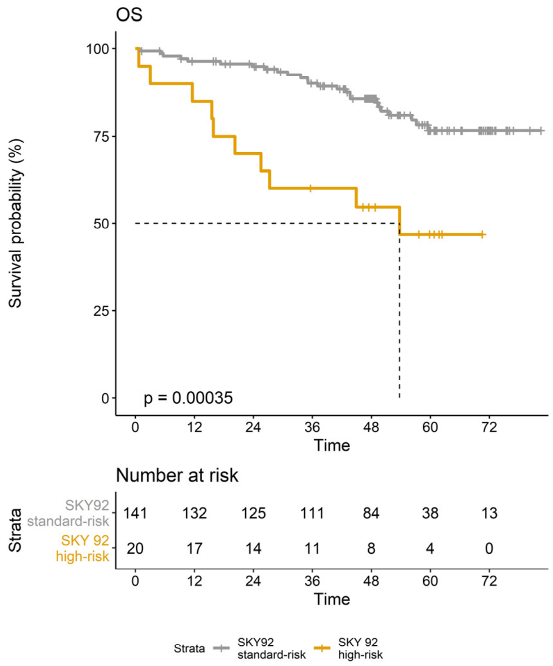

Supplementary Figure 5

Kaplan-Meier plots of molecular risk groups defined by absence of any high-risk marker, presence of a single genetic marker, presence of either double-hit or SKY92 high-risk or combined double-hit and SKY92 high-risk for A) PFS B) OS landmarked from HDMEL+ASCT consolidation.

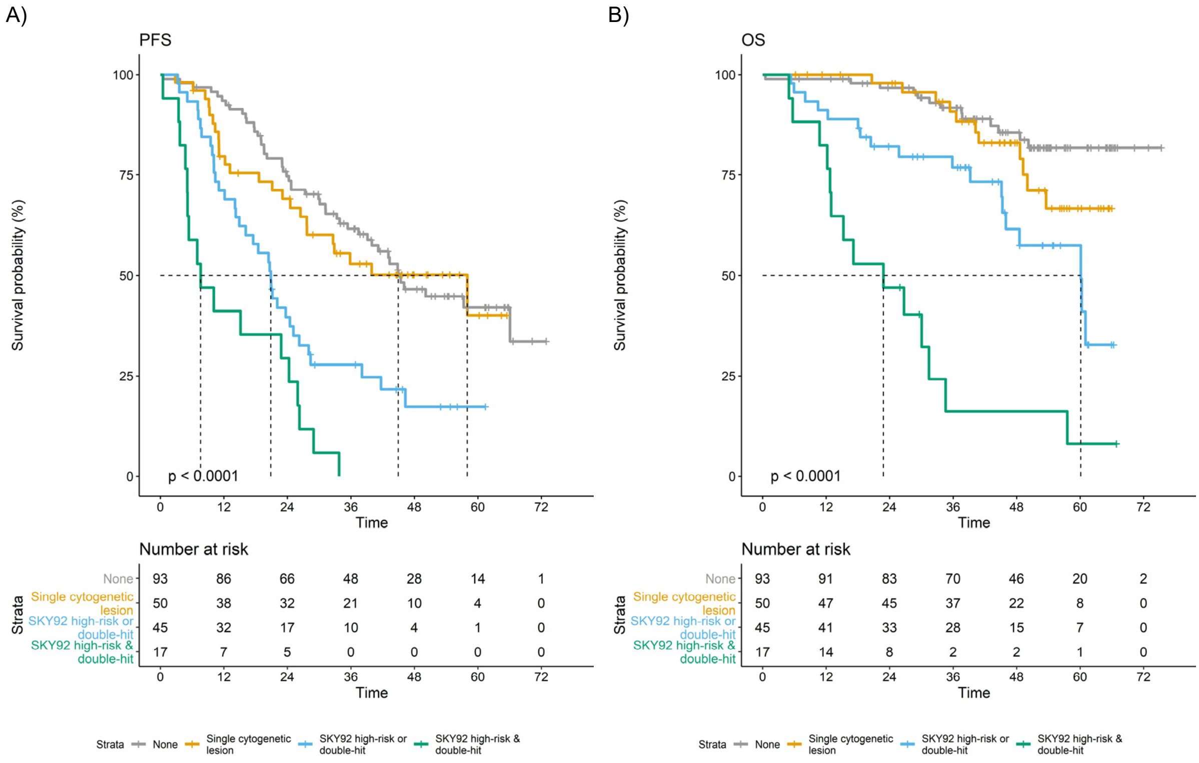

Supplementary Figure 6

Kaplan-Meier plots of molecular risk groups defined by absence of any high-risk marker, presence of a single genetic marker, presence of either double-hit or SKY92 high-risk or combined double-hit and SKY92 high-risk for A) PFS B) OS from maintenance randomization. Kaplan-Meier plots for molecular risk groups of patients that underwent maintenance lenalidomide or observation only. Landmarked analysis from Lenalidomide maintenance randomization, showing lenalidomide vs. observation for sub-groups with C) no genetic or gene expression high-risk marker D) single genetic high-risk marker E) double-hit or SKY92 high-risk F) double-hit and SKY92 high-risk.

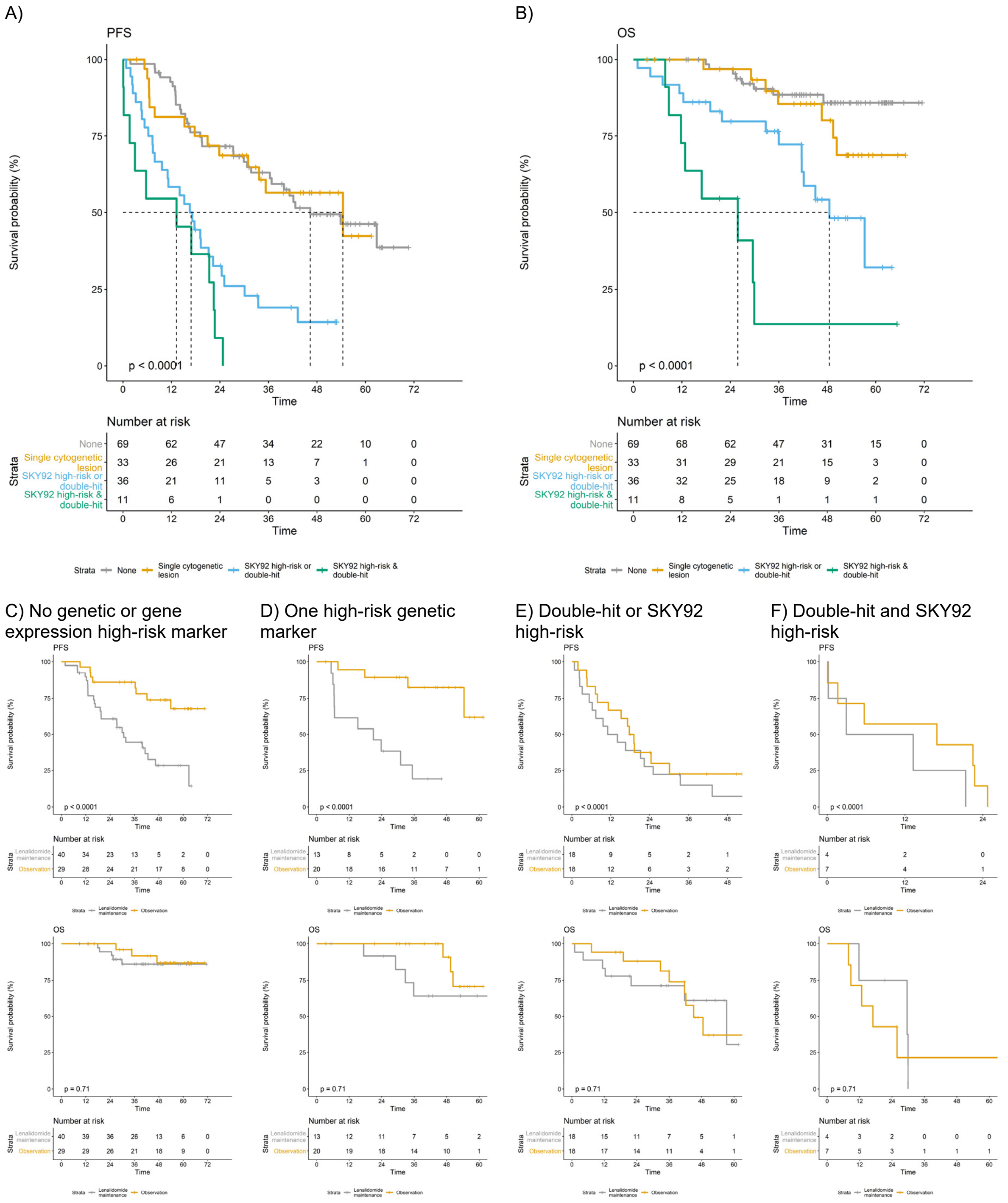

Supplementary Figure 7

Kaplan-Meier plots of intensive treatment arm (ITT; high dose melphalan+ASCT) patients with combined double-hit and SKY92/EMC92 ultra high-risk vs. those without from two phase 3 trials, NCRI Myeloma XI (A, B) and MRC Myeloma IX (C, D) from time of induction randomization.

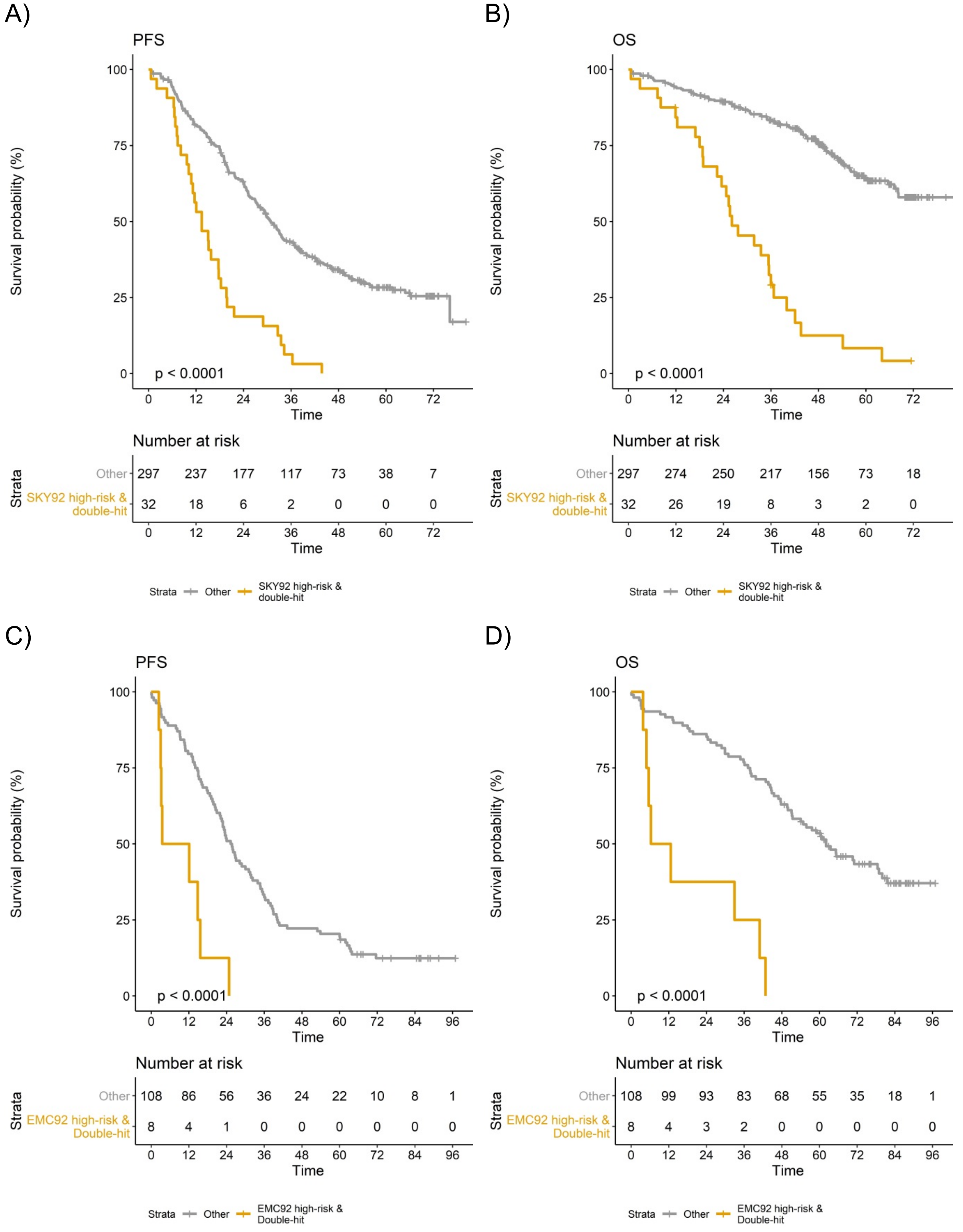

Supplementary Figure 8

Kaplan-Meier plots of NCRI Myeloma XI patients as per diagnostic ISS risk scores from induction randomisation: A) PFS, B) OS.

A)

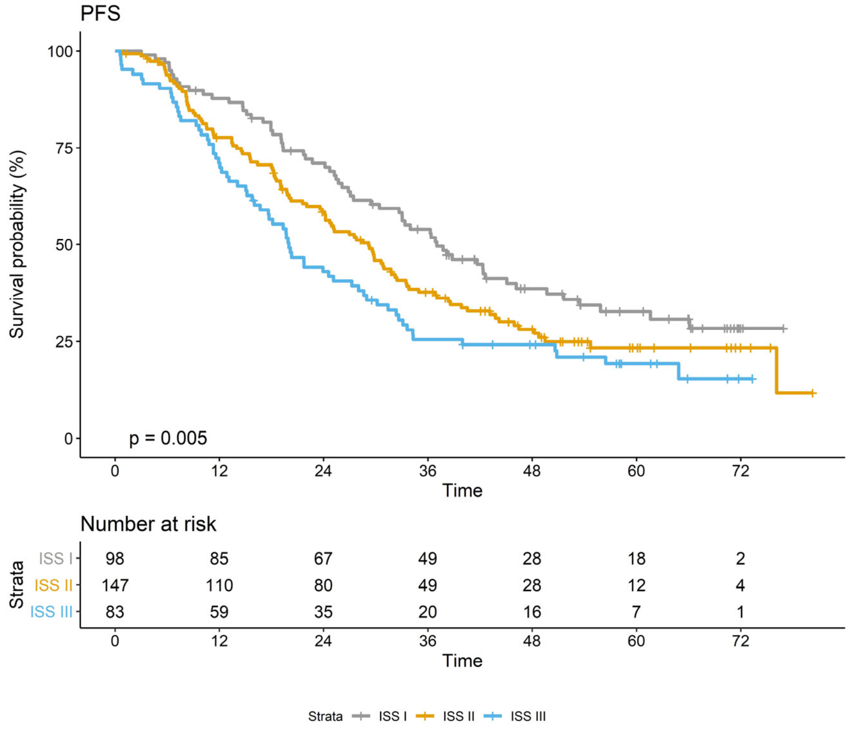

B)

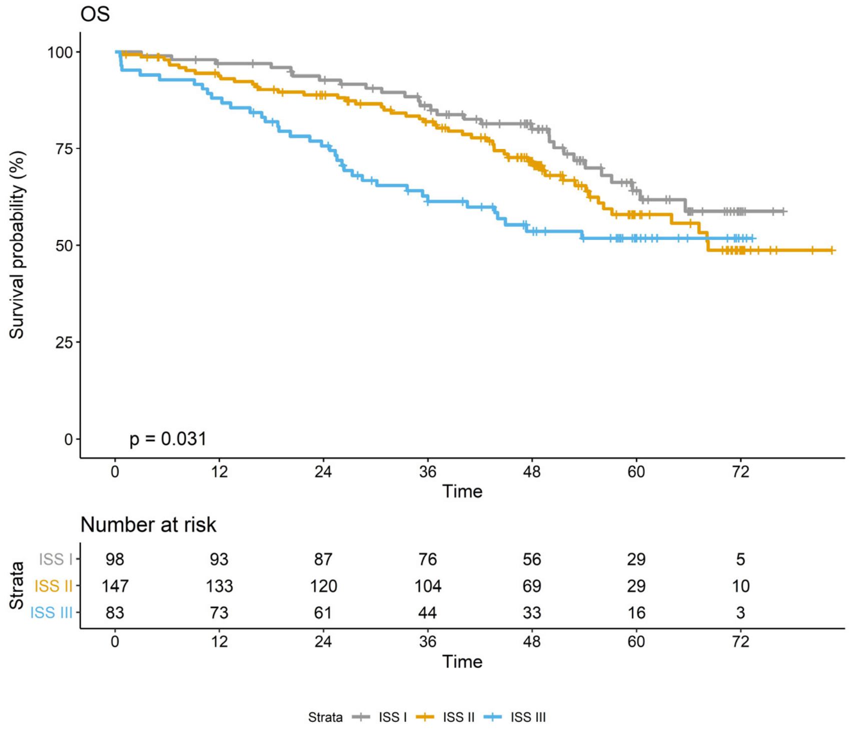

Supplementary Figure 9

Proportion of patients with ISS status for groups with A) no, single or double-hit genetics and patients with B) absence or presence of SKY92 high risk. LDH values at presentation for patients with C) no, single or double-hit genetics and patients with D) absence or presence of SKY92 high risk.

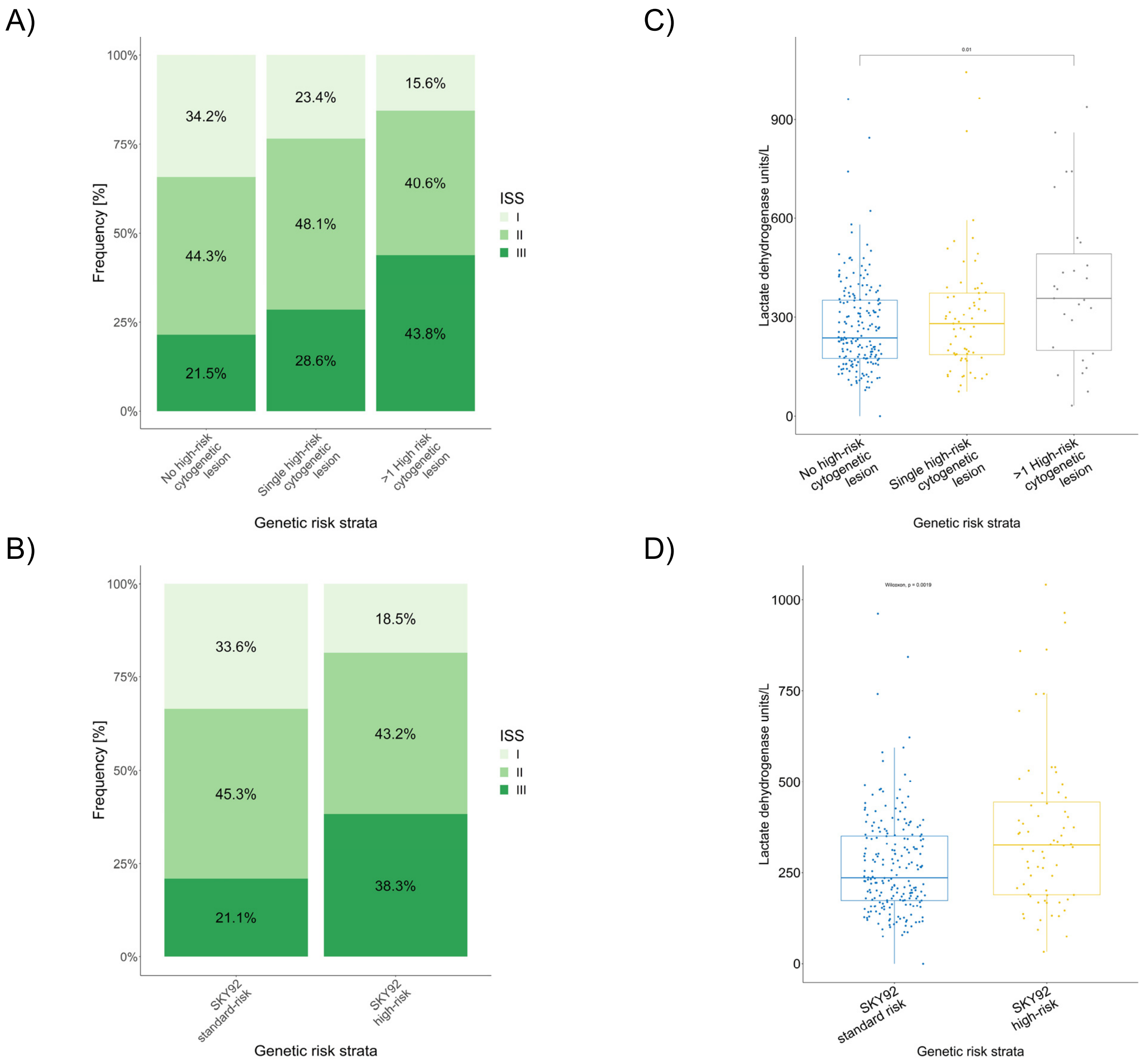

Supplementary Figure 10

A) ISS B) LDH and C) platelet count at diagnosis as per defined risk strata. Venn diagram showing number and overlap of patients with ISS 3, SKY92 high-risk and any chromosomal risk marker. D) Venn diagram of absolute number of and overlap between patients with ISS 3, SKY92 high-risk and/or chromosomal risk markers.

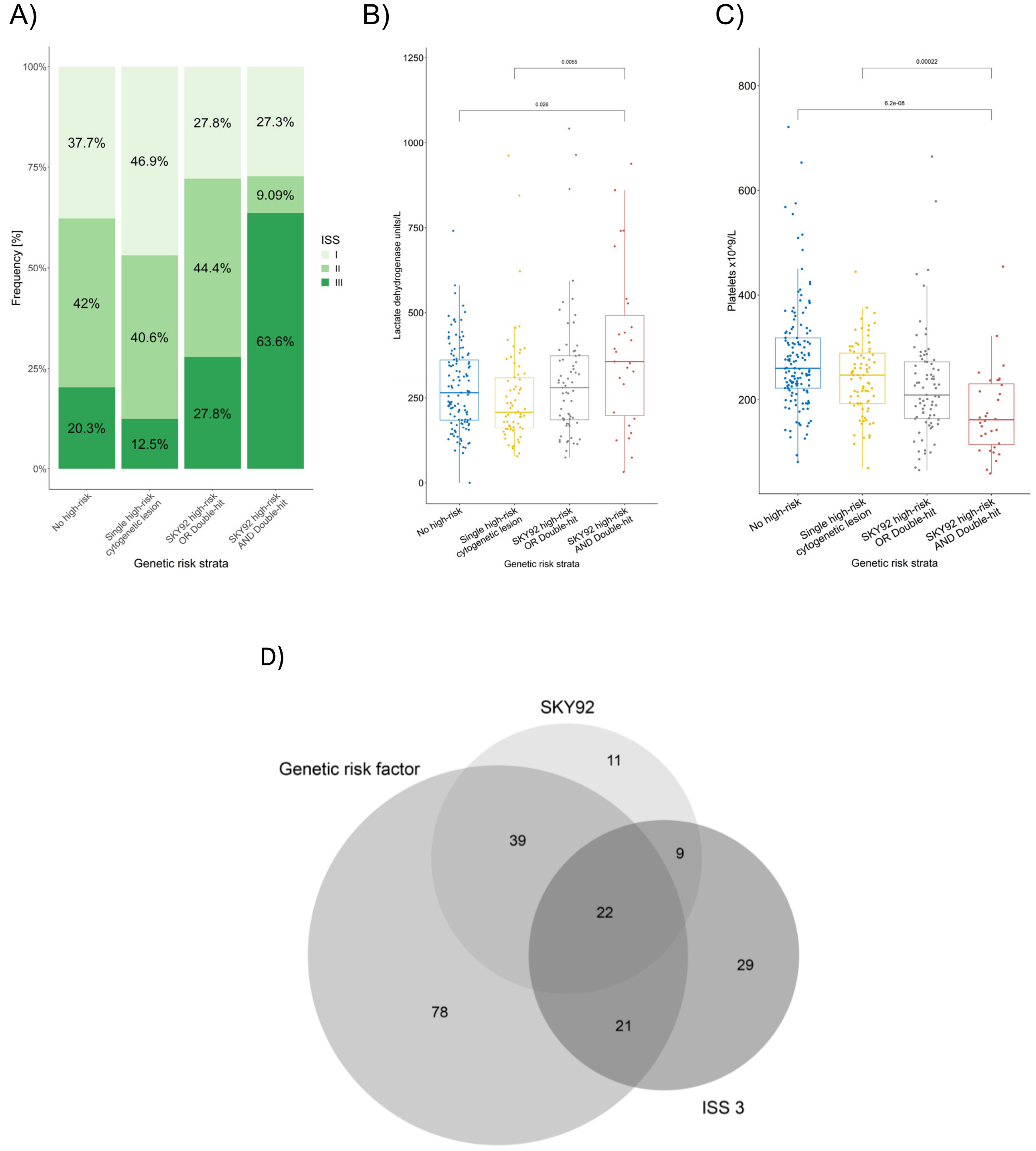

Supplementary Figure 11

A) Correlation plot of quantitative scores for published risk and/or biological GEP signatures. Color coding correlation coefficient. Abbreviations: HMCL7- 7 gene prognostic signature from MM cell line study, CIGNECS = chromosome instability signature, CNTI = centrosome index, HZDCD = homozygous cell death signature (MIX signature), IFM-15 – prognostic signature by IFM Myeloma 99 clinical trial, PI = Proliferation signature, UAMS70 and UAMS80 = 70 and 80 gene signature by UAMS, EMC92 = prognostic signature HOVON-65/GMMG-HD4. B) Dot plot of quantitative scores for EMC92 and UAMS GEP70 signatures.

A)

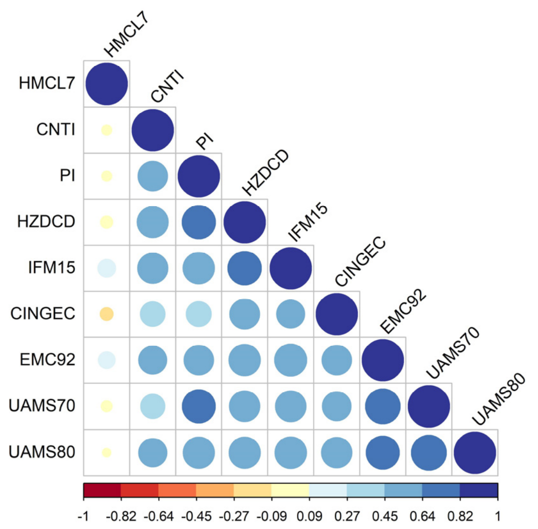

B)

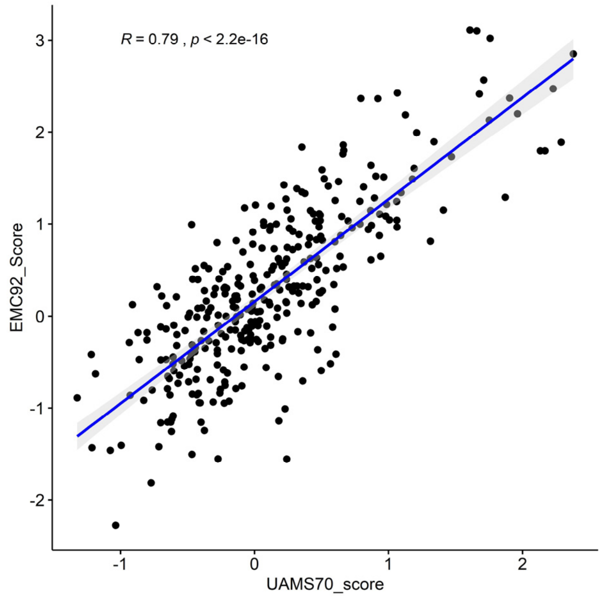

Supplementary Figure 12

EMC92 quantitative score plotted for groups with A) normal, gained or amplified chromosome 1q B) normal, heterozygous or homozygous deletion 17p.

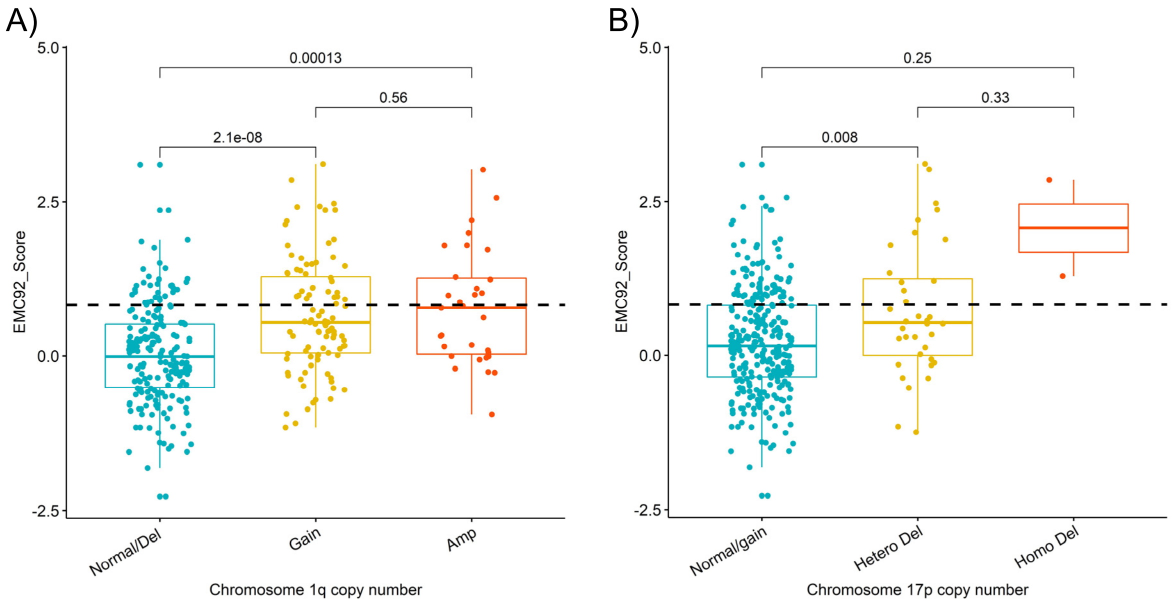

Supplementary Figure 13

Bar plot of quantitative EMC92 continuous scores in quantitative order. Yellow colored bars were positive for the presence of a high risk translocation (t(4;14), t(14;16) or t(14;20) and blue colored bars did not have these. All patients above 0.827 were considered high risk as per EMC92 criteria.

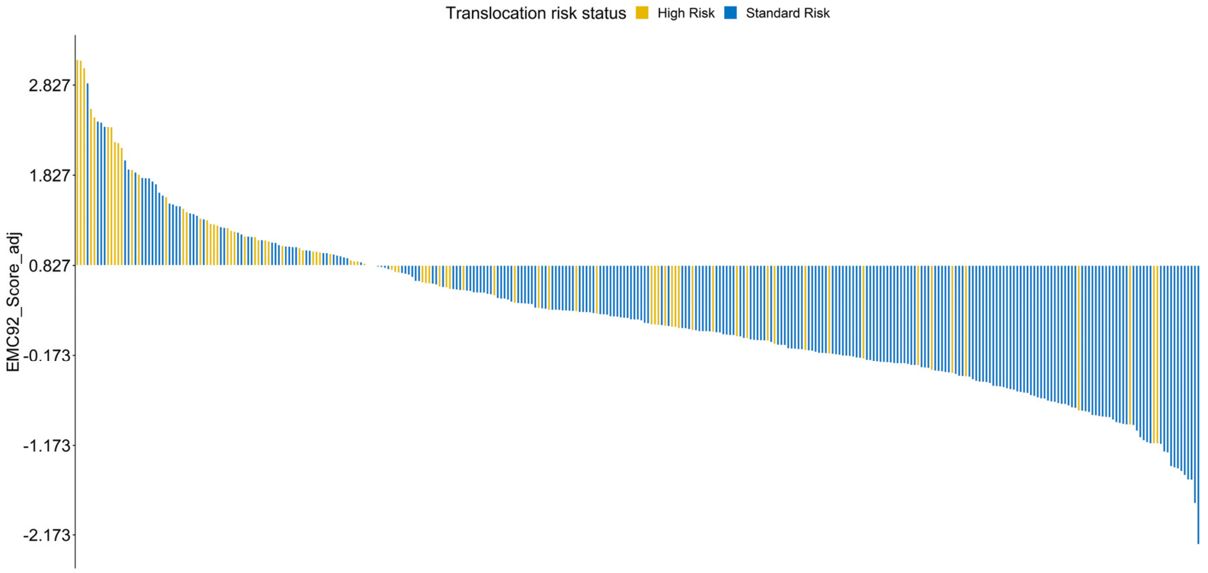

Supplement: Supplementary file 1 — Supplementary Information [file 41375_2020_750_MOESM1_ESM.pdf]
